# Signal regulatory protein α dynamically mediates macrophage polarization facilitated alleviation of ischemic diseases

**DOI:** 10.1186/s13578-024-01325-2

**Published:** 2024-12-20

**Authors:** Haiyi Liu, Yonghui Yuan, Takerra K. Johnson-Stephenson, Chenyang Jing, Mingchao Zhang, Jun Huang, Ke Zen, Limin Li, Dihan Zhu

**Affiliations:** 1 State Key Laboratory of Natural Medicines, School of Life Science and Technology, China Pharmaceutical University, Nanjing, Jiangsu China; 2 State Key Laboratory of Pharmaceutical Biotechnology, Jiangsu Engineering Research Center for MicroRNA Biology and Biotechnology, Nanjing University School of Life Sciences, Nanjing, Jiangsu China; 3 National Clinical Research Center of Kidney Diseases, Jinling Hospital, Nanjing University School of Medicine, Nanjing, Jiangsu China; 4 Cardiovascular Research Institute, Morehouse School of Medicine, Atlanta, GA USA

**Keywords:** Macrophage polarization, Sirpα, Ischemic diseases, Time-dependent

## Abstract

**Background:**

macrophage-targeting therapy of ischemic disease has made progress in clinic trial. However, the role and underlying mechanism of pro-inflammatory or anti-inflammatory polarized macrophages in modulating ischemic diseases remain incompletely understood.

**Results:**

here we examine the effect of pro-inflammatory (LPS) and anti-inflammatory (IL-4) macrophage on ischemic diseases in a mouse ischemic hindlimb and heart model, and identify that signal regulatory protein α (Sirpα) modulates macrophage polarization induced angiogenesis via promoting phagocytosis or activating HIF1α nucleus relocation in macrophages, respectively. More importantly, the therapeutic effect of polarized macrophages is controlled by Sirpα in a time-dependent manner. Downregulation of macrophage Sirpα at the early-stage or upregulation of macrophage Sirpα at the late-stage of ischemic disease enhances the therapeutic effect. In contrast, increasing Sirpα at the early-stage or decreasing it at the late-stage leads to failure of inducing ischemic disease resilience. Mechanistically, we find that signal transducer and activator of transcription 3 and 6 (Stat3 and Stat6) mediate downregulation (pro-inflammatory polarization) or upregulation (anti-inflammatory polarization) of Sirpα, respectively.

**Conclusion:**

Our results reveal that dynamic regulation of macrophage by Sirpα plays a critical role in alleviating ischemic diseases.

**Supplementary Information:**

The online version contains supplementary material available at 10.1186/s13578-024-01325-2.

## Background

Ischemic diseases remain the most common cause of mortality and morbidity in modern society. To address these issues, the science of therapeutic angiogenesis has been evolving for decades [1, 2]. The importance of immune cells, particularly macrophages, in treating ischemic diseases has been emphasized [3–7]. Macrophages are one of the most critical immune effector cells; their remarkable plasticity gives them the ability to respond to environmental cues [8]. Traditionally, depending on their distinct functions, macrophages can be divided into two groups, pro-inflammatory and anti-inflammatory/tissue repair facilitating [9]. Pro-inflammatory macrophages are driven by pro-inflammatory cytokines and/or heterogeneous stimuli that exhibit pro-inflammatory, bactericidal, and phagocytic functions. In contrast, anti-inflammatory macrophages, generated in response to IL-4 or IL-10, secrete anti-inflammatory cytokines and growth factors and help tissue repair [10].

It has been shown that the presence of IL-4 polarized macrophages at the ischemic site is essential for successful tissue repair and resilience of the disease [11, 12]. However, there is a big gap in our understanding of the underlying mechanisms of macrophage paradoxical polarization along with promoting angiogenesis during the treatment of ischemic diseases. Although anti-inflammatory (tissue repair) macrophages are needed for successful angiogenesis in ischemic diseases, the role of pro-inflammatory macrophages in the clearance of apoptotic cells due to ischemia-induced hypoxia has also been recently discussed [13]. Pro-inflammatory macrophages promote angiogenesis in mouse myocardial infarction by efficiently cleaning apoptotic debris. Build up of apoptotic debris induces continuous inflammation and prevents the microenvironment at the ischemic site shifting from a pro-inflammatory to an anti-inflammatory/tissue repair scenario.

Signal regulatory protein alpha (Sripα) is a signaling protein expressed on myeloid leukocytes, including neutrophils, dendritic cells, monocytes, and macrophages [14]. Sirpα regulates macrophage activation, including phagocytosis and cytokine secretion, by­ recruiting and phosphorylating SHP-1 and SHP-2 [15, 16]. Moreover, by ligating to the universally expressed CD47, Sirpα endows macrophage's ability to distinguish between self and none-self [17]. In most cases, apoptotic cells lose CD47 on their surface, which shut down CD47-Sirpα signaling and stimulated macrophage phagocytic activity [18]. However, ischemic disease shifts the normal paradigm. Due to the hypoxic microenvironment, apoptosis-induced downregulation of CD47 is suppressed, thus, inducing the activation of the CD47-Sirpα pathway and attenuating the clearance of hypoxia-associated apoptotic cells [19]. Uncleaned apoptotic cells induced persistent inflammation and led to the inhibition of angiogenesis. Downregulation of Sirpα in the macrophages can rescue macrophages from hypoxia elevated CD47 hijack [20]. Although, the role of Sirpα in tumor treatment has been discussed [21], its possible role in ischemic diseases is largely unknown.

In clinical application, dosage, and time-point of drug use play an equal and critical role in disease treatment [22], however, the latter is often overlooked in research. According to clinical research, ischemic disease can be generally divided into two stages –early-stage and late-stage. The emergency of ischemia induces hypoxic conditions in tissue at early-stage, causes apoptosis of cells, which elevate the pro-inflammatory immune response [23, 24]. The pro-inflammatory microenvironment in the ischemic tissue roses macrophage pro-inflammatory polarization thus accelerating the clearance of apoptotic cells. At the late-stage, promoting tissue repair is the main theme, given credit to the shift of microenvironment, the dominant of macrophage is anti-inflammatory/tissue repair phenotype, which triggers and facilitates the angiogenesis in ischemic tissue. In our study, Sirpα, is downregulated at the early-stage but upregulated at the late-stage of ischemic hindlimb and heart of mice, contributed to macrophage polarization-induced attenuation of the ischemic syndrome. Losing Sirpα facilitates macrophages to clean apoptotic cells due to the ischemia at the early-stage, and regaining Sirpα at the late-stage guarantees tissue repair by inducing the anti-inflammatory cytokines and growth factors secretion. Artificial manipulation of the level of Sirpα would lead to the deterioration of ischemic-induced tissue damage. Under pathological conditions, even without any treatment, ischemia would induce the losing and regaining of Sirpα, but at a prolonged rate. Accelerating the loss and regain of Sirpα at the exact time-point promises a better recovery from ischemic disease.

### Methods

## Sex as a biological variable

Our study examined male and female animals, and similar findings are reported for both sexes.

## Animals

The age of 6–8 weeks old C57BL/6 J and Sirpα knockout mice were from Model Animal Research Center, MARC (Jiangsu, China). Before the experiment, all animals were reared at pathogen-free conditions at room temperature 25 ± 2 ℃, relative humidity 65 ± 2%, and a 12 h light / dark cycle in the Laboratory Animal Center of China Pharmaceutical University. The study followed the institutional ethical guidelines for animal experiments. For surgeries (hind limb and heart), isoflurane (concentration of 1%−4% in oxygen, once) was used to anesthetize mice. Carbon dioxide (CO_2_) overdose was employed for the euthanasia of mice (CO_2_ fill rate of 30–70% of the chamber volume per minute for 5 min).

## Cell culture

The Mouse cardiac microvascular endothelial cells (mCMVECs) were purchased from Cellbiologics (Chicago, IL). The cells were cultivated in a microvascular endothelial cell Growth Medium (Cellbiologics) containing 100 units/ml of penicillin, 100 μg/ml of streptomycin at 37 °C, 5% CO_2_. The medium was changed to fresh medium every 2–3 days.

## Macrophage isolation, adoption and depletion

Macrophage isolation: adductor muscles were processed as previously described [51], and the cell pellet was resuspended in D-hanks containing 0.5% BSA and 2 mM EDTA. Briefly, adductor muscles were minced in a digestion solution (Roche Diagnostics, IN, USA). The solution was then incubated at 37ºC for 1 h and was terminated by PBS with 10% FBS. The mixture was then filtered with a 70 µm cell strainer and centrifuged at 250 g for 10 min. The pellet was washed with PBS and centrifuged at 250 g for 10 min. Anti-F4/80 coated MACS beads (Miltenyi, Germany) were used to sort macrophages.

Macrophage adoption: macrophages sorted from the adductor muscles of C57 mice or Sirpα^−/−^ mice were resuspended in 0.9% saline. 1 × 10^5^ macrophages were injected into the adductor muscles of ischemic mice at indicated times.

Macrophage depletion: clodronate-liposomes (YEASEN, China) were employed to deplete macrophages in the mice and were processed as previously described [52]. Briefly, all mice received 200 μL clodronate liposomes through intraperitoneal injection 24 h pre-surgery to deplete macrophage. From 24 h post-surgery, 50 μL clodronate liposomes were injected into the mice adductor muscles every 3 days to maintain the macrophage depletion.

## Flow cytometry analysis for macrophages

Macrophages isolated from adductor muscles of ischemic mice that received various treatment, were resuspended and blocked for 30 min in D-hanks with 2% BSA. The cells were then incubated with 1:500 diluted FITC-anti-F4/80, or PE-anti-CD206 and PE-anti-CD86, (BioLegend; San Diego, CA) for 45 min (room temperature and protected from light). The cells were then washed with D-hanks twice and resuspended in D-hanks with 2% BSA for flow cytometry analysis to determine the phenotype of the macrophages. The F4/80 positive cells were gated and defined as macrophage, and then the percentage of CD206 or CD86 positive cells in all macrophages was measured. The cells incubated with FITC-Isotype or PE-Isotype (BioLegend) were used as the isotype control.

## Western blot analysis

Cells were washed with PBS twice and lysed. Protein samples were extracted and then separated on a 4%−12% precast Bis–Tris gel (Thermo Fisher Scientific; Waltham, MA). Later proteins were transferred to polyvinylidene difluoride (PVDF, Rancho Palos Verdes, USA) membranes. The membrane was blocked for 1 h in TBST (with 5% non-fat milk) and incubated with primary antibodies overnight at 4 °C. Proteins were evaluated by using primary anti-Sirpα, anti-Stat3, anti-phosphorylated Stat3, anti-Stat6, anti-phosphorylated Stat6 and anti-HIF1α (Cell Signaling, MA) antibodies. Anti-GAPDH and anti-PCNA antibodies were purchased from Thermo Fisher Scientific. Membranes were washed with TBST 3 times (total 45 min). The washed membranes were incubated with second antibodies for 1 h, at room temperature. Enhanced chemiluminescence reagents were used to illuminated protein and ImageQuant ™ LAS 4000 Luminescent Image Analyzer (GE Healthcare; Chicago, IL) was used to expose protein bands.

## Tunel staining

TUNEL staining was performed using the TUNEL Apoptosis Detection Kit (YEASEN, China), according to the manufacturer’s protocol. All images were obtained using a fluorescence microscope (ECLIPSE Ti2, Nikon, Japan). The results of immunofluorescence pictures were analyzed by using an ImageJ software (Media Cybernetics).

## ELISA assays

The conditioned medium (CdM) or serum was collected to evaluate the concentration of cytokines or growth factors. Macrophages isolated from the adductor muscles of the mice that received various treatments were cultured in macrophage serum-free medium (Thermo Fisher Scientific) for 24 h. Then, the medium was collected, and the cytokines and growth factors were evaluated. Levels of certain cytokines and growth factors including IL-1α, IL-6, GM-CSF, IL-10, TGF-β, M-CSF, VEGF and bFGF by using ELISA kits (R&D Systems). The procedures for the cytokine and growth factor assays were carried out according to the manufacturer's instructions. The Molecular Devices Spectramax spectrophotometer (Marshall Scientific, Hampton, NH) was employed to measure the concentration of cytokines.

## Phagocytosis assays

Mouse cardiac microvascular endothelial cells were incubated under hypoxic conditions for 3 days or cultured with FBS deprived medium for 48 h to induce apoptosis. The percentage of apoptotic cells was evaluated by flow cytometry. The apoptotic cells were washed with PBS twice and labeled with pHrodo-Red (Thermo Fisher Scientific). Then the 5 × 10^4^ cells were mixed with 5 × 10^4^ macrophages (isolated and sorted from mouse adductor muscles) to determine the phagocytotic capacity. After, being incubated for 2.5 h at 37 °C, the macrophages were washed three times with ice-cold D-hanks and stained with FITC rat anti-mouse CD11b antibody (Biolegend) for 45 min at 4 °C. Then the cells were harvested and examined by flow cytometry. Non-apoptotic cells received pHrodo-Red treatment were served as a negative control.

## RNA isolation and RT-qPCR for miRNAs

Total RNA was extracted from cells using TRIzol Reagent (Thermo Fisher Scientific) according to the manufacturer’s instructions. Reverse transcription and quantitative real-time PCR were performed using the TaqMan miRNA assay system (Thermo Fisher Scientific) according to the manufacturer’s instructions. The relative miRNA levels were normalized to endogenous U6 small nuclear RNA for each sample.

## Hindlimb ischemia (HLI) model

All animal experiments in this study were approved by the Institutional Animal Care and Use Committee of the China Pharmaceutical University and complied with the NIH guidelines for the care and use of laboratory animals. The femoral artery was ligated on the left hindlimb of C57BL/6 J mice (WT) and Sirpα knockout mice (Sirp^−/−^, the Jackson Laboratory, Bar Harbor, ME) at age 6–8 weeks according to our previous report [12]. The mice were subjected to intramuscular injection in the left adductor muscle with PBS or 100 ng LPS or 50 ng IL-4 immediately after ligation. The same intramuscular injections were performed at the surgery site 3 and 7 days post-surgery. For time-point study, same amount of LPS or IL-4 was intramuscular injected into adductor muscle at 0 and 4 days (early-stage) or 8 and 12 days (late-stage) post surgery. Blood perfusion on the plantar surface of the hind paws was measured immediately before and after surgery and 3, 7, 14, and 21 days post-surgery using laser speckle imaging (LSI, Perimed AB, Järfälla, Sweden). The left to right ratio (L/R) was used to represent the relative blood perfusion rate at the left hindlimb for each animal (n = 10). The mice were euthanized 21 days after the surgery. The left adductor muscle and gastrocnemius muscle were collected for macrophage isolation, immunoblotting and immunohistochemistry analyses.

## Myocardial infarction (MI) model

C57BL/6J mice (10–12 weeks old) were kept anesthetized on a heating pad in a supine position by inhalation of 2% isoflurane via a face mask. Surgery procedures were performed as Gao et al. described [53]. Briefly, a skin cut, 10–12 mm in length, was made over the left chest, and a purse-string suture was made around the incision. The fourth intercostal space was opened after dissection and retraction of the pectoral major and minor muscle. The heart was immediately and gently “popped out” through the opening. The left anterior descending (LAD) coronary artery was ligated. PBS or LPS or IL-4 was intramyocardially injected in the infarct border area by using a 32 G microsyringe (Hamilton, Reno, NV). The heart was allowed to retract in the thoracic cavity followed by manual evacuation of air and closure of the skin opening with the previously placed purse-string suture. Intravenous injection of PBS or LPS or IL-4 was performed through tail vein at 7 days post-surgery. The mice were euthanized 28 days post-surgery. Hearts were collected for immunoblotting, as well as Masson’s staining and immunohistochemistry analyses after paraffin-embedding and transversely sectioning.

## Echocardiography

Echocardiography was performed twice for each mouse at 1 day before and 28 days after the LAD coronary artery ligation. Mice were anesthetized with inhalation of isoflurane. Transthoracic 2-dimensional images were obtained using a high-resolution echocardiography system HP5500 (Hewlett-Packard, San Jose, CA) equipped with a 15-MHz transducer. Two-dimensional guided M-mode was then used to measure left ventricle (LV) end-systolic diameter (LVESD) and LV end-diastolic diameter (LVEDD) at the mid-ventricular level. The percentage of LV fractional shortening (LVFS%) was calculated as ((LVEDD − LVESD)/LVEDD × 100%).

## Masson's staining

MI was evaluated by using a trichrome stain (Masson) kit (Millipore Sigma Aldrich) on cardiac sections according to the manufacturer’s instructions. The normal myocardium showed red while the infarcted myocardium showed blue due to a collagen fibril formation. Infarct sizes of LV were evaluated as described in a previous report [54]. Briefly, a LV myocardial midline was drawn at the middle between the epicardial and endocardial surfaces by using an ImageJ software. The percentage of the arc length occupied by infarcted myocardium in the LV circumference was calculated as the infarct sizes of the myocardial section. The average percentage from four layers at 1 mm intervals was used to represent the infarct sizes of LV.

## Immunohistochemistry

The muscle or heart sections were stained with the primary anti-CD31 antibody (1:500, BD Biosciences) and counterstained with DAPI as described previously [12]. The regions containing the most intense CD31^+^ areas of neovascularization were chosen for quantification. Five hotspots per section and 3 sections per muscle were analyzed at 400 × magnification. ImageJ software was used to measure CD31^+^ areas in each hotspot.

## Cell proliferation

MCMVECs were quiesced with endothelial basal medium/1% FBS for 24 h and incubated in 5 µmol/L fluorescent carboxyfluorescein succinimidyl ester (CFSE) solution (green) from a CellTrace CFSE Cell Proliferation Kit (Thermo Fisher Scientific) at 37 °C for 20 min. The cells were then washed and incubated in each of the various CdM from macrophages, buffered with an equal volume of endothelial basal medium/1% FBS. The same medium was changed on day 2. The cells were harvested at day 4 and subjected to analysis using a flow cytometer with 488-nm excitation and emission filters appropriate for fluorescein.

## Migration assay

MCMVECs were treated with various CdM from macrophages for 24 h. The treated mCMVECs were then seeded in a 24-well insert plate (BD Biosciences; San Jose, CA) and incubated at 37 °C for 24 h. Cell nuclei were stained with Hoechst 33,342 (Thermo Fisher Scientific). The cells that migrated to the lower side of the inserts were counted as described in our previous report [12].

## Tube formation assay

MCMVECs were treated with various CdM from macrophages. The treated mCMVECs were seeded on precoated growth factor-reduced Matrigel (BD Biosciences) and incubated with endothelial basal medium/1% FBS at 37 °C for 4 h. The cells were then stained with Calcein AM (Thermo Fisher Scientific), and tube formation was visualized using fluorescence microscopy. The total vessel length was calculated using AngioTool v.2 software [12].

## Transduction of recombinant lentivirus

The procedures practiced here followed the National Institutes of Health guidelines for recombinant DNA research. To knockdown or overexpress specific protein in the cells, the lentivirus based protein knockout or overexpression systems were employed. All recombinant lentiviruses used in this study were purchased from the amsbio (Abingdon, UK). For transduction, macrophages were incubated with the Lenti/siStat3 (siStat3), Lenti/siStat6 (siStat6) or Lenti/siSirp⍺ (IL-4-Sirp⍺) at a multiplicity of infection (MOI) of 5, to deplete Stat3 or Stat6 levels in the cells. Lent/siCont (siCont) was used as a control. For Sirp⍺ overexpression, macrophages were incubated with the Lenti/ Sirp⍺ (LPS + Sirp⍺) to overexpress Sirp⍺ in the cells.

## Statistics

All values are showed as the means ± SDs. All experiments were repeated at least 4 times (n = 4) unless otherwise noted. One-way ANOVA followed by Tukey multiple range test was performed to analyze all data. Only *p* < 0.05 was considered significant.

## Study approval

All animal experiments in this study were approved by the Institutional Animal Care and Use Committee of China Pharmaceutical University and complied with the NIH guidelines for the care and use of laboratory animals.

## Results

### LPS and IL-4 promoted angiogenesis in mouse hindlimb ischemia model

**Fig. 1:**
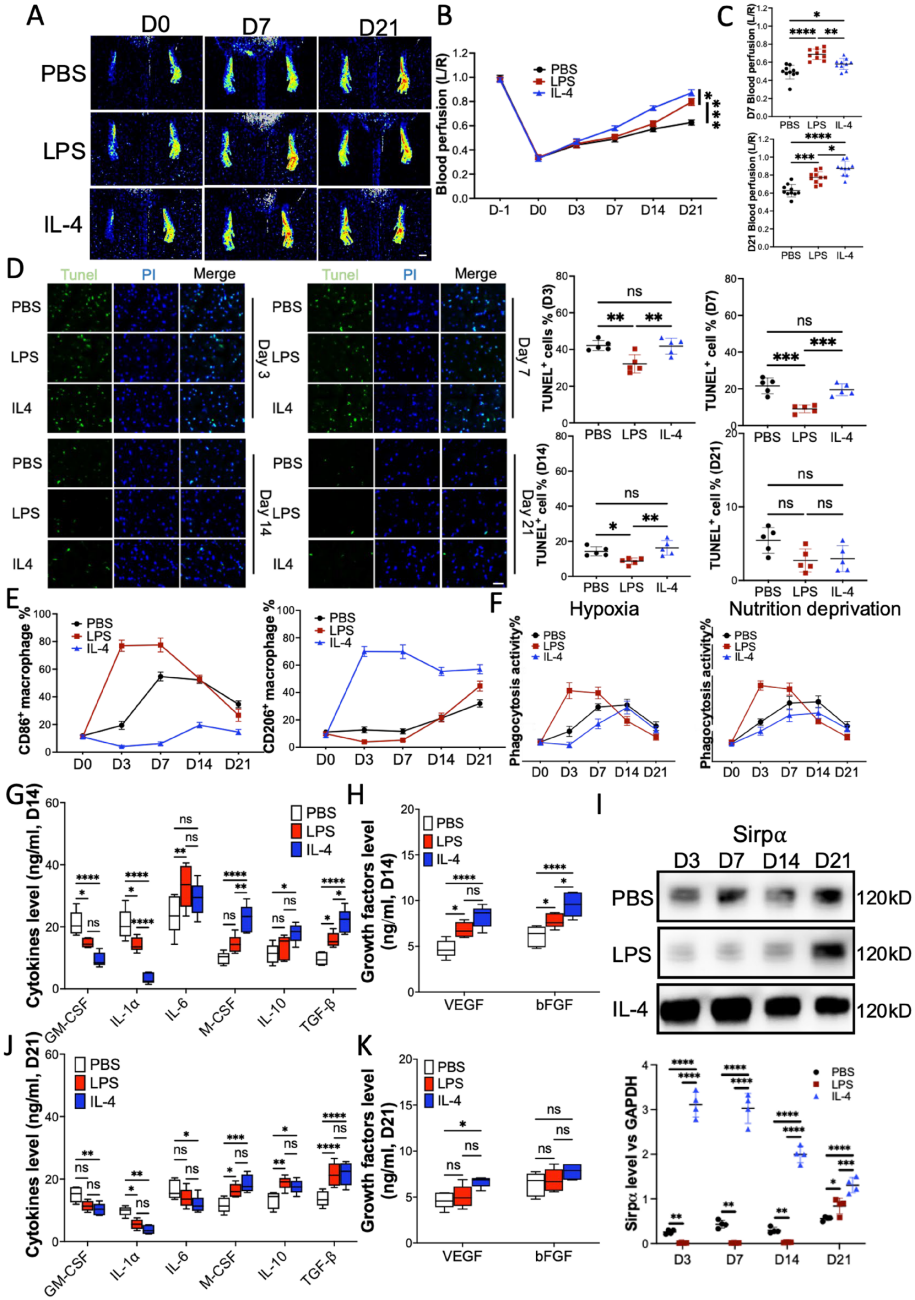
LPS and IL-4 promoted angiogenesis and macrophage polarization in a mouse HLI model. After left artery ligation, the mice received PBS, LPS and IL-4 intramuscular injection on day 0, 3 and 7 post-surgery (n = 10). **A**–**C**, Laser speckle data showing the relative level of blood perfusion in the hind paws on the indicated days (scale bar: 1000 µm, **A** and **B**). Quantitative analyses of the laser speckle images showing the left/right ratio of plantar blood perfusion (D7 and D21, **C**). **D** The percentages of dead cells in gastrocnemius muscles from ligated hindlimb on D3, D7, D14, and D21 post-surgery (Tunel Assay, scale bar: 100 µm). **E** Macrophage were isolated from adductor muscles and the surface markers, CD86 (pro-inflammatory polarization marker), and CD206 (anti-inflammatory polarization marker) were checked using flow cytometry. Total 5000 cells were gated and analyzed. **F** The phagocytosis of pHrodo Red-labeled hypoxia or nutrition deprivation induced apoptotic mCMVEC by the macrophages isolated from adductor muscles of the mice that received various treatment. Total 5000 cells were analyzed. **G**, **H** The cytokines (**F**) and growth factors (**G**) secreted from the macrophages that were collected from adductor muscles of mice 14 days post-surgery, were measured using ELISA. **I**, **J** The secreted cytokines (**H**) and growth factors (**I**) from the were collected from adductor muscles of mice 21 days post-surgery. **K** The level of Sirpα in the macrophages collected from adductor muscles of the mice that received various treatment on indicated days post-surgery (n = 3). Data is analyzed using one-way ANOVA followed by Tukey multiple range test and expressed as mean ± SD of n = 5, unless specified. *p < 0.05, **p < 0.01, ***p < 0.001 and ****p < 0.0001. ns, non-significant

Both LPS-induced pro-inflammatory polarized macrophage and IL-4-induced anti-inflammatory polarized macrophage are known to promote angiogenesis in mouse hindlimb ischemia and myocardial infarction models [13, 25]. In our study, LPS or IL-4 was injected into the adductor muscle of hindlimb ischemia mice to evaluate the pro-angiogenic effect (sFigure 1). Compared to the mice that received PBS injection, the mice that received LPS or IL-4 presented a higher blood perfusion level at the paw from day 7post-surgery (Fig. 1A −1 C). The results of immunohistochemistry analysis showed that the vascular endothelial cell marker CD31 in the gastrocnemius muscles was elevated by LPS or IL-4 treatment (sFigure 2). Terminal deoxynucleotidyl transferase dUTP Nick-End Labeling (TUNEL) assay has been designed to detect cell death in gastrocnemius muscles from the mice receiving various treatments. Our results confirmed that the LPS-treated hindlimb ischemia mice reserved lower dead cells in gastrocnemius muscles from day 3 and 7 post-surgery (Fig. 1 D). To explore the phenotype of macrophages at the ligation site, the macrophages in the adductor muscle were harvested and then analyzed by flow cytometry. The results indicated that LPS treatment aroused pro-inflammatory polarized macrophages at early stages (day 3 and 7) compared to PSB treatment, while IL-4 treatment promoted anti-inflammatory polarization of macrophages (Fig. 1 E and sFigure 3). Further, the phagocytosis assay demonstrated that macrophages from LPS-treated mice phagocyted more hypoxia or nutrition deprivation (without FBS) induced apoptotic cells than macrophages from IL-4 or PBS-treated mice (Fig. 1F). Cleaning apoptotic debris quicker endows the ability to turn over the pro-inflammatory microenvironment to an anti-inflammatory microenvironment faster at ischemic sites. Consistent with this finding, cytokine and growth factor assay showed pro-inflammatory cytokines in the serum of LPS-treated mice (sFigure 4A – 4C) or secreted by macrophages collected from the same mice were downregulated significantly from day 14 post-surgery (Fig. 1G and 1H). More interestingly, although IL-4 treatment preserved the highest level of anti-inflammatory cytokines and pro-angiogenic growth factors on day 3 and 7 post-surgery (sFigure 5A – 5C), the situation changed from day 14 post-surgery (Fig. 1G and 1H), due to the clearance of dead cells LPS treatment presented similar level of anti-inflammatory cytokines when compared to IL-4 treatment. Furthermore, there was no significant difference in anti-inflammatory and pro-angiogenic growth factors between LPS and IL-4 treatment on day 21 post-surgery (Fig. 1I, J). These results suggested that inducing pro-inflammatory cytokines and promoting anti-inflammatory cytokines secretion may be the reasons for LPS or IL-4-induced pro-angiogenic effect respectively. Moreover, immunoblotting showed that the level of Sirpα, which mediates macrophage activation, was downregulated in mice that received LPS on day 14 or earlier and restored later, compared to PBS treatment. IL-4 treatment upregulated Sirpα levels from day 3 post-surgery until day 21 (Fig. 1K). Our previous study demonstrated that down-regulation of Sirpα in the macrophage resulted in the up-regulation of phagocytosis activity [15]. In addition, the raised Sirpα level, accompanied by the higher anti-inflammatory cytokines and growth factors, suggested a link between them. Moreover, macrophage depletion was employed to further determine the role of macrophage in the LPS or IL-4 induced angiogenesis in hindlimb ischemic mice. The mice received 200 μL clodronate liposomes through intraperitoneal injection 24 h pre-surgery to deplete macrophage (sFigure 6A), PBS was used as a control. Blood perfusion results showed macrophage depletion attenuated the pro-angiogenic effect of LPS or IL-4 (sFigure 6B). Altogether, our results confirmed that not only IL-4 but also LPS promoted angiogenesis in the mouse hindlimb ischemia model. IL-4 induced elevation of anti-inflammatory cytokines and pro-angiogenic growth factors which has been verified to promote angiogenesis [26]. LPS-induced cleaning of apoptotic cells made the turnover of the micro-environment from pro-inflammation to anti-inflammation possible, thus facilitating angiogenesis.

## LPS or IL-4 facilitate time-dependent promotion of angiogenesis in mouse ischemia hindlimb

**Fig. 2:**
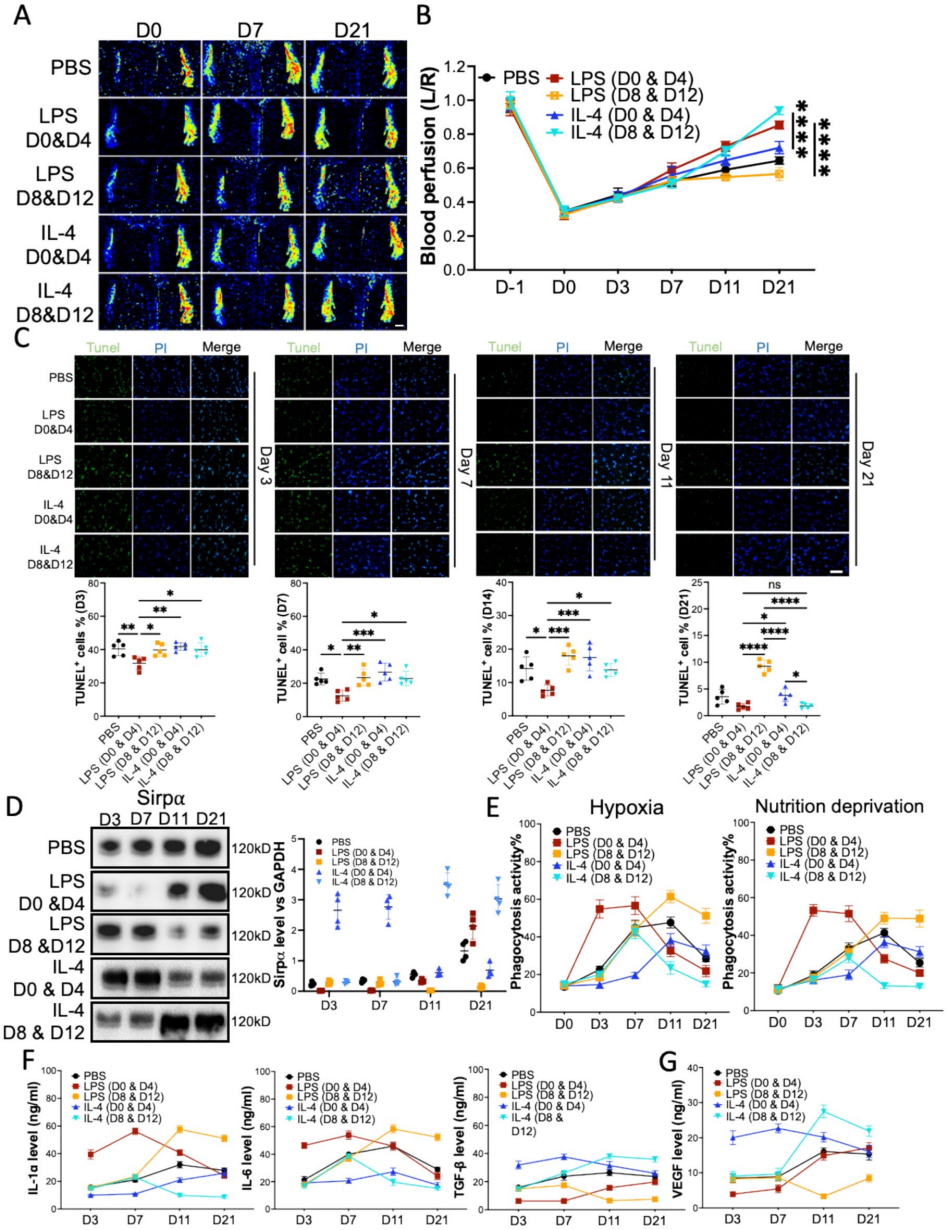
LPS and IL-4 promoted angiogenesis in mouse hindlimb ischemia in a time-dependent manner. After left artery ligation, the mice received PBS, LPS, and IL-4 intramuscularly injections at various times. **A**, **B** Laser speckle data showing the relative level of blood perfusion in the hind paws of mice that received various treatments on the indicated days (scale bar: 1000 µm). **C** The percentages of dead cells in gastrocnemius muscles from the mice on D3, D7, D14, and D21 post-surgery, scale bar: 100 µm. **D** The level of Sirpα in the macrophages collected from adductor muscles of the mice (n = 3). **E** The phagocytosis of pHrodo Red-labeled hypoxia or nutrition deprivation induced apoptotic mCMVECs by the macrophages from adductor muscles of the mice. Total 5000 cells were gated and analyzed. **F**, **G** IL-1α, IL-6, TGF-β (**F**), and growth factors (**G**) secreted from the macrophages that were collected from adductor muscles of the mice on the indicated days, were measured using ELISA. PBS, LPS D0 & D4 or IL-4 D0 & D4: the mice received PBS, LPS or IL-4 intramuscular injection on day 0 and day 4 post-surgery. LPS D8 & D12 or IL-4 D8 & D12: the mice received LPS or IL-4 intramuscular injection on day 8 and day 12 post-surgery. Data is analyzed using one-way ANOVA followed by Tukey multiple range test and expressed as mean ± SD of n = 5, unless specified. *p < 0.05, **p < 0.01 and ***p < 0.001****p < 0.0001. ns, non-significant

Traditionally, LPS promotes pro-inflammatory macrophage generation, while IL-4 induces macrophage anti-inflammatory polarization [27]. To further study the pro-angiogenic effect of LPS and IL-4, LPS or IL-4 was injected into ligated sites of mice at the early-stage (day 0 and 4) or late-stage (day 8 and 12) post-surgery, respectively (sFigure 7). Our results showed that the hindlimb ischemic mice that received LPS at the early-stage or IL-4 at the late-stage presented with better blood perfusion (Fig. 2A and 2B, sFigure 8A) and increased angiogenesis (larger CD31^+^ area, sFigure 8B) compared to the mice that received LPS at the late-stage or IL-4 at the early-stage, respectively. In-depth analyses of dead cells in the gastrocnemius muscles demonstrated that early-stage LPS treatment resulted in the lowest number of dead cells. However, the mice that received LPS at the late-stage presented the highest number of dead cells on day 21 (Fig. 2C). Immunoblotting results further demonstrated that although LPS downregulated Sirpα levels both in early-stage and late-stage on day 21 post-surgery, early-stage LPS treatment preserved a higher level of Sirpα. However, IL-4 treatment demonstrated different results. Early-stage IL-4 appliance resulted in a lower Sripα level from day 14 compared to the late-stage IL-4 treatment (Fig. 2D). Phagocytosis activity analysis of macrophages from the adductor muscles confirmed that compared to the late-stage LPS or early-stage IL-4 treatment, the macrophages from early-stage LPS or late-stage IL-4 injected mice phagocyted more apoptotic cells (Fig. 2E). Cytokines and growth factors assay showed the early-stage LPS treatment induced a significant decrease of pro-inflammatory cytokines and an increase of anti-inflammatory cytokines as well as growth factors from day14 post-surgery (Fig. 2F and 2G, sFigure 9), which contributed to the LPS-induced angiogenesis, compared to the late-stage LPS administration. Although IL-4 treatment induced downregulation of pro-inflammatory cytokines more or less retarded the clearance of hypoxia-induced apoptotic cells in gastrocnemius muscles at the early-stage (Fig. 2C and 2E), the remarkable upregulation of anti-inflammatory cytokines and growth factors promoted angiogenesis compared to the PBS treatment (Fig. 2F and 2G, sFigure 10). More interestingly, due to the inflammatory microenvironment at the early stage, the mice that received IL-4 injection at the late stage showed better blood perfusion and more angiogenesis when compared to the mice that received early-stage IL-4 infusion (Fig. 2B). Altogether, our results indicated that although both LPS and IL-4 promoted angiogenesis in the mouse hindlimb ischemia model, the time point for the implementation was crucial. Early-stage administration of LPS induced quicker apoptotic cell clearance or late-stage dosing of IL-4 induced upregulation of anti-inflammatory cytokines and growth factors contributed to the alleviation of mouse hindlimb ischemia.

## Loss-gain SIRPα in the macrophages mediated LPS- or IL-4-induced angiogenesis

**Fig. 3:**
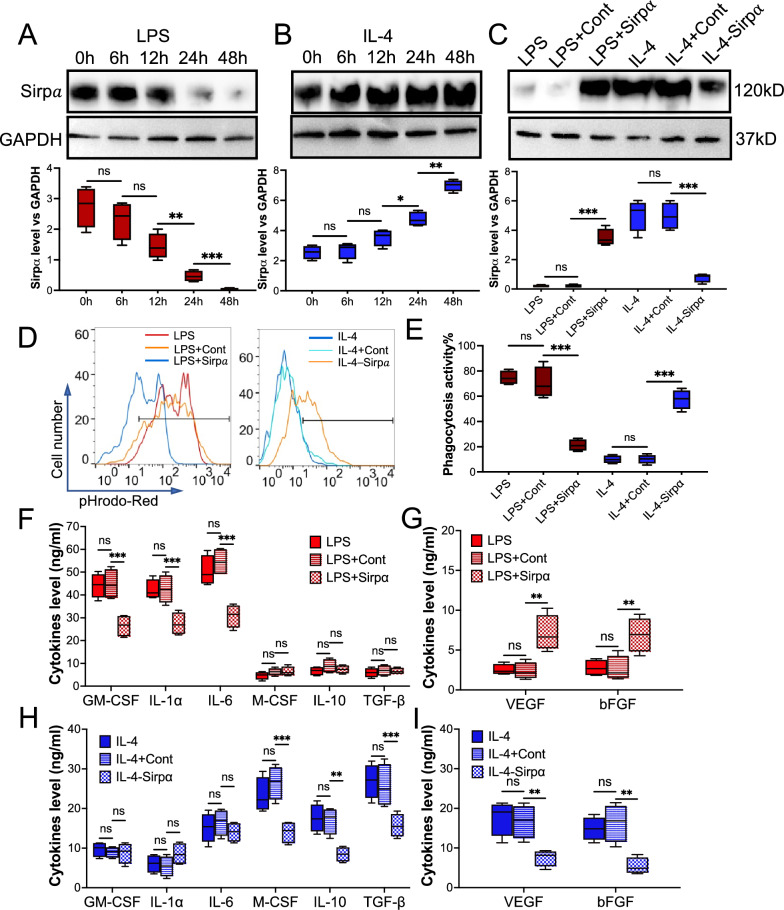
Restored or knockdown Sirpα in the macrophage that received LPS or IL-4, respectively, caused function disorder of the macrophage. Macrophages were isolated from the adductor muscles of wild-type mice. Sirpα was increased or decreased in macrophages that pre-treated with 30 ng/ml LPS or IL-4 for 48 h by Lentivirus (LPS + Sirpα: LPS treatment with Sirpα overexpression or IL-4-Sirpα: IL-4 treatment with Sirpα downregulation), respectively. Lentivirus control was used as the control (LPS + Cont: LPS treatment with Lentivirus control and IL-4 + Cont: IL-4 treatment with Lentivirus control). **A**, **B** The level of Sirpα (n = 3) in the macrophages that received LPS (**A**) or IL-4 (**B**). **C** The level of Sirpα (n = 3) in the pre-treated macrophages that were incubated with Lentivirus to manipulate Sirpα expression. **D**, **E** The phagocytosisof pHrodo Red-labeled apoptotic mCMVECs by the macrophages were measured by flow cytometry (**D**, total 5000 cells were analyzed). Quantification of the phagocytosis percentages (**E**). **F**, **G** The cytokines (**F**) and growth factors (**G**) secreted from the macrophages that received LPS, LPS + Cont or LPS + Sirpα, were measured using ELISA. **H**, **I** The secreted cytokines (**H**) and growth factors (**I**) from the macrophages that received IL-4, IL-4 + Cont or LI-4-Sirpα. Data is analyzed using one-way ANOVA followed by Tukey multiple range test and expressed as mean ± SD of n = 5, unless specified. *p < 0.05, **p < 0.01 and ***p < 0.001. ns, non-significant

Our and others’ previous results indicated that Sirpα mediated macrophage activation[28]. Here we designed Sirpα loss-gain experiments to determine the role of Sirpα in macrophage-induced angiogenesis. Immunoblotting confirmed that LPS induced downregulation of SIRPα in the macrophage (Fig. 3A), and IL-4 presented an opposite behavior (Fig. 3B). A lentivirus-based Sripα loss or gain system was employed to modulate the level of Sirpα in the macrophage (Fig. 3C). The LPS treated macrophages were incubated with lentivirus based Sirpα overexpression system to upregulate Sirpα level (LPS + Sirp⍺), and lentivirus based Sirpα knockdown system was employed to downregulate Sirpα level in IL-4 treated macrophages (IL-4-Sirp⍺). Phagocytic activity analyses showed that increasing Sirpα in the LPS treated macrophage dampened the apoptotic cell clearance while downregulating Sirpα in the IL-4-treated macrophages enhanced its phagocytic activity (Fig. 3D and 3E). To further study the underlying mechanisms of Sirpα in promoting angiogenesis, we evaluated the levels of pro- and anti-inflammatory cytokines and growth factors in the conditioned medium collected from macrophages received various treatments. Our results revealed that although upregulation of Sirpα in LPS-treated macrophages did not affect the level of pro- and anti-inflammatory cytokines, the level of growth factors was upregulated significantly (Fig. 3F and 3G).

In vitro angiogenesis assays, including endothelial cell proliferation (sFigure 11), migration (sFigure 12), and tube-formation (sFigure 13), were performed to evaluate the pro-angiogenic effect of macrophages that received various treatments. Our results showed that overexpressed Sirpα in the LPS-treated macrophages increased the pro-angiogenic effect, while knocking down Sirpα in the macrophages attenuated the IL-4 facilitated proangiogenic effect. Moreover, downregulation of Sirpα in the IL-4 treated macrophages dramatically decreased anti-inflammatory cytokines and growth factors (Fig. 3H and 3I).

Our results showed that upregulating Sirpα in LPS-educated macrophages increases endothelial cell proliferation, migration, and tube formation while downregulating SIRPα in IL-4 treated macrophages decreases macrophage pro-angiogenic effect.

## LPS- or IL-4 failed to promote angiogenesis in the Sirpα knockout mice

**Fig. 4:**
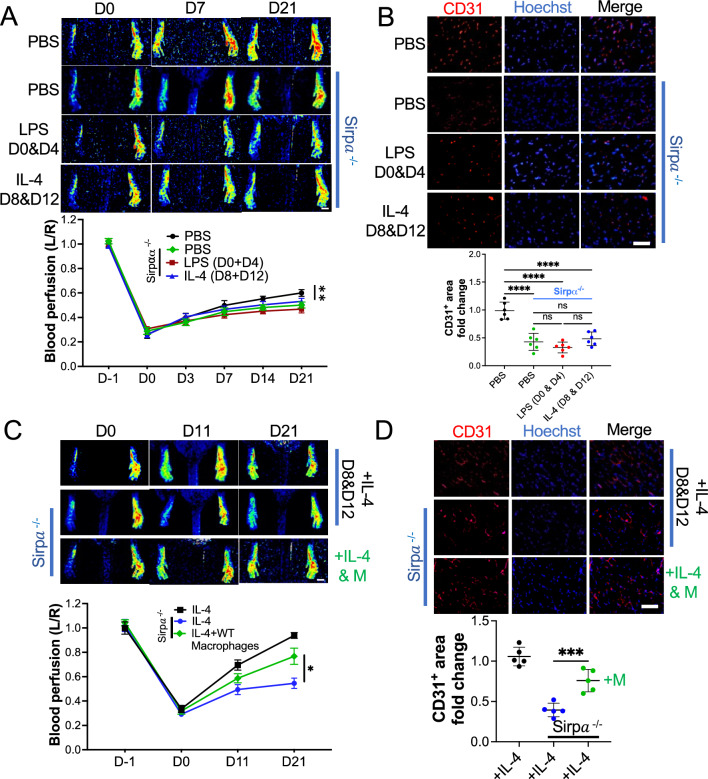
Sirpα contributed the pro-angiogenic effect of LPS or IL-4 in vivo. **A** After left femoral artery ligation, PBS, LPS or IL-4 was intramuscularly injected into Sirpα knockout mice (Sirpα^−/−^) on indicated days post-surgery, WT mice with PBS injection were used as a control (n = 6). Laser speckle data showing the relative level of blood perfusion in the hind paws of mice that received various treatments on the indicated days (scale bar: 1000 µm). **B** The mice were euthanized 21 days post-surgery. The sections of the gastrocnemius muscle from the ligated side were subjected to immunohistochemistry analysis for CD31 and counterstained with Hoechst 33342 (scale bar: 100 µm, n = 6). Quantification of the CD31^+^ area. The CD31^+^ area on the slide from the mouse administered with PBS was set to 1. **C** Macrophages isolated from WT mice were pre-treated with IL-4 for 48 h and then were injected into ischemic Sirpα^−/−^ mice, ischemic WT mice or Sirpα^−/−^ mice that received IL-4 injection were used as controls (n = 5). Laser speckle data showing the relative level of blood perfusion in the hind paws of mice that received various treatments on the indicated days (scale bar: 1000 µm). **D** The sections of the gastrocnemius muscle from the ligated side of the mice were subjected to immunohistochemistry analysis for CD31 and counterstained with Hoechst 33342 (scale bar: 100 µm, n = 5). Quantification of the CD31^+^ area. The CD31^+^ area on the slide from the WT mouse administered with IL-4 was set to 1. Sirpα^−/−^: Sirpα knock mice, PBS: PBS injection on day 0 and day 4 post-surgery, LPS D0 & D4: LPS injection on day 0 and day 4 post-surgery, IL-4 D8 & D12: IL-4 injection on day 8 and day 12 post-surgery. + IL-4 D8 & D 12: IL-4 injection on day 8 and day 12 post-surgery, + IL-4 & M: IL-4 pre-treated WT macrophage injection on day 8 and day 12 post-surgery. Data is analyzed using one-way ANOVA followed by Tukey multiple range test and expressed as mean ± SD. *p < 0.05, **p < 0.01, ***p < 0.001 and ****p < 0.0001. ns, non-significant

To further study the role of Sirpα in LPS and IL-4-induced alleviation of hindlimb ischemia, the Sirpα knockout (Sirpα^−/−^) mice were sacrificed. All Sirpα^−/−^ mice received hindlimb artery ligation to create an ischemia model, the wild-type mice received the same surgery and were used as a control. On day 0 and day 3, the Sirpα^−/−^ mice received PBS, LPS, or IL-4 *in-situ* injection, WT mice received PBS injection were used as a control. Our results demonstrated that the pro-angiogenic effect of LPS and IL-4 were attenuated in the Sirpα^−/−^ mice, the blood perfusion was lower in Sirpα^−/−^ mice even than PBS treated WT mice from day 7 post-surgery (Fig. 4A and sFigure 14A). There was no significant difference of blood perfusion between LPS and IL-4 treated Sirpα^−/−^ mice. Although tunnel assay indicated that knockout Sirpα facilitated the obligation of apoptotic cells in the gastrocnemius muscles (sFigure 14B), without the restriction of Sirpα, over-provoked macrophages induced severe anemia in Sirpα^−/−^ mice, which was validated by a blood hemoglobin assay (sFigure 14C). Interestingly, flow cytometry analyses showed that knockout Sirpα did not change the polarization of macrophages, LPS-induced pro-inflammatory polarization (CD86^+^), while IL-4 promoted anti-inflammatory polarization of macrophage (CD206^+^) from ischemic Sirpα^−/−^ mice (sFigure 15A). In addition, phagocytosis assay showed macrophages from Sirpα^−/−^ mice were highly activated, when compared to the WT mice (sFigure 15B). The cytokines and growth factors assay revealed that although Sirpα knockout did not affect the LPS-induced pro-inflammatory cytokines and IL-4-induced anti-inflammatory cytokines secretion (sFigure 16A), the growth factor level was downregulated significantly (sFigure 16B). The angiogenesis in the gastrocnemius muscles was also downregulated in Sirpα^−/−^ mice compared to the WT mice that received PBS (Fig. 4B), which further emphasized the positive role of Sirpα in promoting angiogenesis. More interestingly, adopting macrophages from WT mice restored blood perfusion in Sirpα^−/−^ mice. IL-4 or IL-4 plus macrophages from wild-type mice were injected into the adductor muscles of hindlimb ischemic Sirpα^−/−^ mice on day 8 and day 12; ischemic wild-type mice that received IL-4 were used as a control (sFigure 17). Our results showed the macrophage adoption promoted angiogenesis in ischemic Sirpα^−/−^ mice. The blood perfusion was higher in Sirpα^−/−^ mice with macrophage injection than without ones from day 11 post-surgery (Fig. 4C). The results of immunohistochemistry analysis further confirmed that the vascular endothelial cell marker CD31 in the gastrocnemius muscles was elevated by macrophage adoption in ischemic Sirpα^−/−^ mice (Fig. 4D).

These in vivo study on the Sirpα^−/−^ mice further confirmed that Sirpα is essential for LPS and IL-4 induced angiogenesis.

## LPS and IL-4 regulated Sirpα expression by activating Stat3 and Stat6

**Fig. 5:**
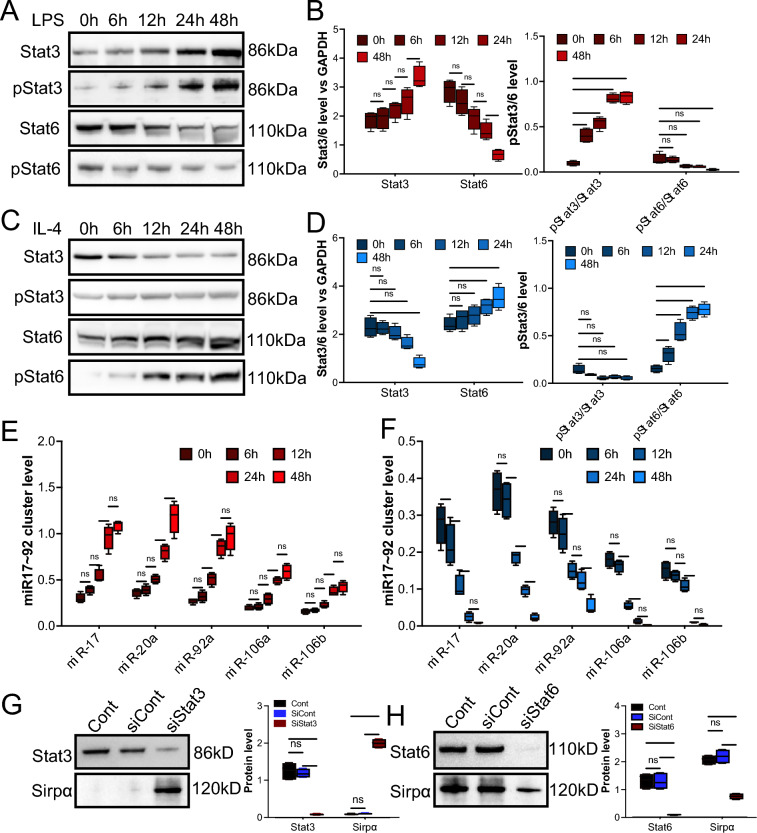
Stat3 or Stat6 mediated to the LPS or IL-4 induced changing of Sirpα. Macrophages from the adductor muscles of wild-type mice were treated with 30 ng/ml LPS or IL-4 at various times. Lentivirus was used to knock down Stat3 (siStat3) or Stat6 (siStat6) in the macrophage. **A**, **B** The level of Stat3, phosphorylated Stat3 (pStat3), Stat6, and phosphorylated Stat6 (pStat6) in the macrophages that received LPS for various time (**A**), and the quantification (**B**, n = 3). **C**, **D** The level of Stat3, pStat3, Stat6, pStat6 in the macrophages that received IL-4 for various times (**C**), and the quantification (**D**, n = 3). **E** and **F** The levels of miR17 ~ 92 cluster in the macrophages that received LPS (**E**) or IL-4 (**F**), were determined by RT-qPCR (n = 4). **G** Stat3 and Sirpα levels in the LPS pre-treated Stat3 knockdown macrophages were examined. GAPDH was used as a control (n = 3). **H** Stat6 and Sirpα levels in the IL-4 pre-treated Stat6 knockdown macrophages were examined. GAPDH was used as a control (n = 3). Data is analyzed using one-way ANOVA followed by Tukey multiple range test and expressed as mean ± SD. *p < 0.05, **p < 0.01, ***p < 0.001 and ****p < 0.0001. ns, non-significant

Although it is known that the activation of Stat3 and Stat6 contributes to the LPS- and IL-4-induced macrophage polarization [29, 30], respectively, the underlying mechanisms of regulation of Sirpα, which are downregulated in LPS-stimulated macrophages but upregulated in IL-4 treated macrophages, are not fully understood. Our previous study demonstrated that the miR-17 ~ 92 cluster post-transcriptionally modulated Sirpα expression. It has been reported that Stat3 positively regulates the miR-17 ~ 92 cluster. However, the role of Stat6 in Sirpα regulation is not thoroughly investigated. Here, the Lentivirus-based knock-down system was used to determine the role of Stat3 and Stat6 in the Sirpα regulation. Our immunoblotting results verified that LPS treatment increased the ratio of phosphorylated Stat3 to pan Stat3 (Fig. 5A and 5B), and IL-4 induced the increase of phosphorylated Stat6 (Fig. 5C and 5D). Moreover, RT-qPCR confirmed that LPS stimulation promoted the expression of the miR-17 ~ 92 cluster, including miR-17, miR-20a, miR-92a, miR-106a, and miR-106b (Fig. 5E). In addition, our results showed that IL-4 treatment decreased the level of the miR17 ~ 92 cluster (Fig. 5F). Further, by knocking down Stat3 (downregulated by Lentivirus-Stat3 knockdown system) in the LPS-treated macrophage, the upregulation of the miR-17 ~ 92 cluster was reversed (sFigure 18A), and knocking down Stat6 (downregulated by Lentivirus-Stat6 knockdown system) in IL-4-treated macrophages induced upregulation of miR-17 ~ 92 cluster (sFigure 18B). Further, the results from immunoblotting showed that downregulation of Stat3 induced upregulation of Sirpα (Fig. 5G), and knockdown of Stat6 resulted in downregulation of Sirpα (Fig. 5H), which is consistent with RT-qPCR results above.

Together, our results provide evidence that Stat3 and Stat6 contribute to LPS or IL-4-induced modulating of Sirpα through manipulating the level of the miR-17 ~ 92 cluster.

## Downregulation of Sirpα interrupted hypoxia-elevated CD47-induced attenuation of phagocytosis activity

**Fig. 6:**
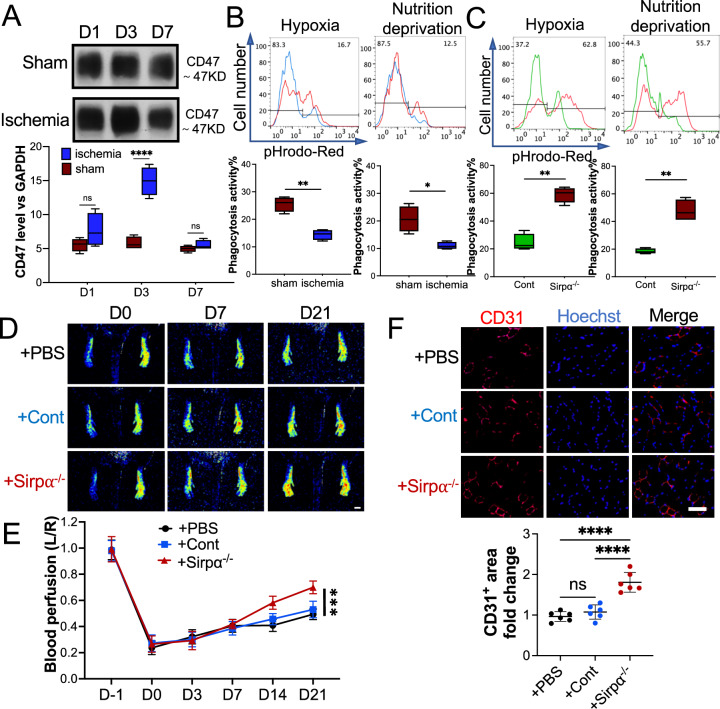
Macrophage from Sirpα knockout (Sirpα^−/−^) mice promoted angiogenesis in ischemic WT mice at early stage. **A** The level of CD47 in the adductor muscles of the mouse that received surgery or sham. GAPDH was used as a control. **B** The phagocytosis of pHrodo Red-labeled apoptotic mCMVECs (hypoxia or nutrition deprivation induced) by the macrophages from the adductor muscles of the mice that received surgery or sham, were tested by flow cytometry. Total 5000 cells were analyzed. **C** The phagocytosis of pHrodo Red-labeled apoptotic mCMVECs by the macrophages from the adductor muscles of the Sirp^−/−^ mice or WT mice (Cont). **D**, **E** The macrophages collected from the adductor muscles of Sirpα^−/−^ mice were transplanted to the ischemic WT mice on day 0 and day 4 post-surgery (early stage), and PBS was used as a control. Laser speckle data showing the relative level of blood perfusion in the hind paws on the indicated days (**D**, scale bar: 1000 µm). Quantitative analyses of the images showing the left/right ratio of plantar blood perfusion (**E**). **F** The mice were euthanized 21 days post-surgery. The sections of the gastrocnemius muscle from the ligated side were subjected to immunohistochemistry analysis for CD31 and counterstained with Hoechst 33342 (**E**, scale bar: 100 µm). Quantification of the CD31^+^ area. The CD31^+^ area on the slide from the mouse administered with PBS was set to 1. Sham: the mice received sham surgery, Ischemia: the mice received femoral artery ligation, + PBS: the ischemic mice received PBS injection, + Cont: the ischemic mice received macrophages from WT mice, + Sirpα^−/−^: the ischemic mice received macrophages from Sirpα^−/−^ mice. Data is analyzed using one-way ANOVA followed by Tukey multiple range test and expressed as mean ± SD of n = 5, unless specified. *p < 0.05, **p < 0.01, ***p < 0.001 and ****p < 0.0001. ns, non-significant

The CD47-Sirpα signaling pathway is employed to distinguish between self and non-self. It has been reported that in responding to hypoxia stress, the level of CD47 in the cells is elevated to avoid obliteration by macrophages[31]. Overexpression of CD47 usually results in the weakened phagocytosis activity of macrophages. However, dampened phagocytosis activity delays the clearance of apoptotic cells, induces persistent inflammation, and results in the deterioration of ischemic diseases[13]. Our results showed that the level of CD47 in the muscles around the ligation site was upregulated at the early-stage post-surgery due to the ischemia microenvironment (Fig. 6A). High-CD47 educated macrophages from these muscles presented much weaker phagocytic activity than macrophages collected from mice that received sham surgery (Fig. 6B). Knocking down Sirpα in the macrophage rescued them from CD47 hijack, and significantly restored the phagocytotic activity of the macrophage (Fig. 6C). To evaluate the impact of the CD47-Sirpα pathway on hindlimb ischemia resilience in vivo, the macrophages collected from adductor muscles of Sirpα^−/−^ mouse were transplanted to the WT mouse post-surgery (on day 0 and day 3 post-surgery,erarly-stage). The Sirpα^−/−^ macrophage transplantation remarkably accelerated blood flow restoration compared to WT-derived macrophages (Fig. 6D). IHC results further demonstrated that Sirpα^−/−^macrophage transplantation induced more angiogenesis in the gastrocnemius muscles (Fig. 6E).

These findings provide a new explanation of LPS-induced pro-angiogenic effects in the hindlimb ischemia model and determines the critical role of losing Sirpα at the early-stage post-surgery in promoting the alleviation of ischemia hindlimb.

## Upregulation of Sirpα promoted the stabilization and relocation of HIF1α to enhance angiogenesis

**Fig. 7:**
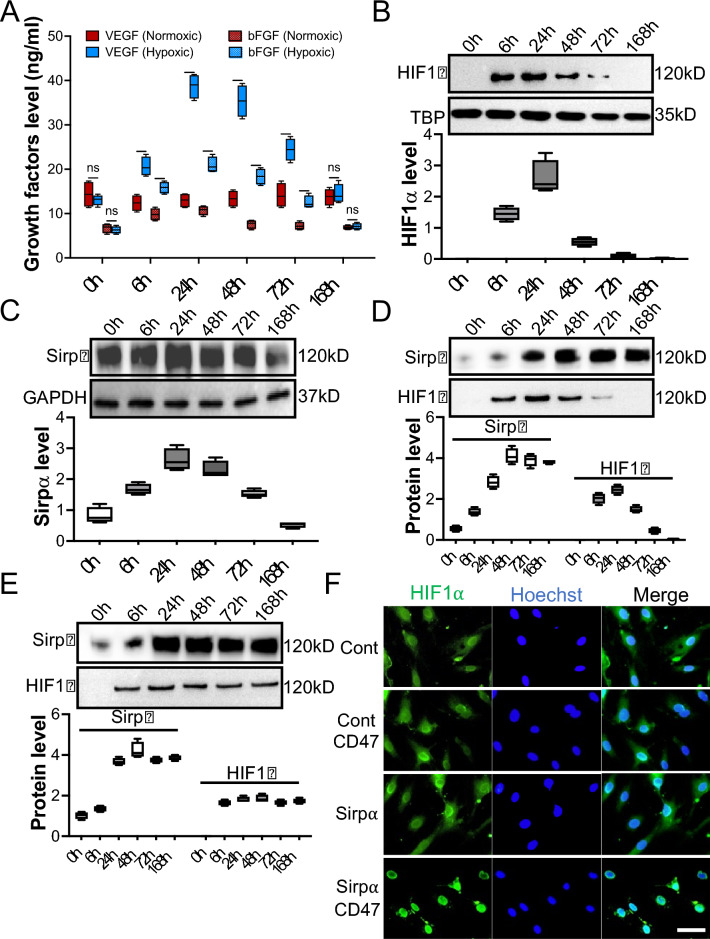
Sirpα promoted the stabilization and relocation of HIF1α. The macrophages collected from adductor muscles of WT mice were pre-treated with 30 ng/ml IL-4 for 48 h to elevate the level of Sirpα and then incubated under hypoxic conditions for 0 to 168 h. **A** The growth factors secreted by the pre-conditioned macrophages were checked using ELISA. **B** The level of HIF1α in the macrophages’ nuclei at various times post-hypoxic pre-conditioning. **C** The level of Sirpα in the macrophages at various times post-hypoxic pre-conditioning. **D**–**F** Lentiviruses were used to overexpress Sirpα in 30 ng/ml IL-4 pre-conditioned macrophages. Then macrophages were incubated under hypoxic conditions for 0 to 168 h with or without the presence of CD47. The level of HIF1α in the Sirpα overexpressed macrophages nuclei without the presence of CD47 at various times post-hypoxic pre-conditioning (**D**). The level of HIF1α in the Sirpα overexpressed macrophages nuclei with CD47 at various times post-hypoxic pre-conditioning, Lentiviruses control-treated macrophages were used as a control (**E**). The nucleus relocation of HIF1α in the macrophages was checked by immunofluorescence (F, n = 4, 168 h post-hypoxic pre-conditioning, scale bar: 50 µm), Cont: Lentiviruses control-treated macrophage without the presence of CD47, Cont CD47: Lentiviruses control-treated macrophage with the presence of CD47, Sirpα: Sirpα overexpressed macrophage without the presence of CD47, Sirpα CD47: Sirpα overexpressed macrophage with the presence of CD47. Data is analyzed using one-way ANOVA followed by Tukey multiple range test and expressed as mean ± SD of n = 3, unless specified. ***p < 0.001, and ****p < 0.0001. ns, non-significant. IC, isotype control

The critical role of HIF1α in treating ischemic diseases has been emphasized[32]. IL-4 has been reported to enhance HIF1α expression in various conditions. However, the underlying mechanisms is debatable. Macrophages were collected from the adductor muscles of WT mice and pre-treated with IL-4 for 48 h, then cultured in normoxia or hypoxia for seven days (168 h, sFigure 19). Surprisingly, although, hypoxia treatment induced elevation of the growth factors of macrophages immediately post-incubation, there were no significant differences in secreted growth factors between normoxic and hypoxic macrophages from 168 h post-incubation (Fig. 7A). Immunoblotting results further revealed that although hypoxia-induced upregulation of HIF1α in nuclei, its level dropped dramatically from 48 h post-hypoxia exposure (Fig. 7B). Interestingly, hypoxia-induced upregulation of Sirpα from 6 h post-hypoxic pre-conditioning was also observed. However, its level paralleled the fluctuation of HIF1α, which declined significantly from 48 h (Fig. 7C). To explain this puzzling phenomenon, Sirpα was overexpressed in hypoxic macrophages using Lentivirus to explore the possible interaction between Sirpα and HIF1α. Beyond our expectation, immunoblotting results showed the level of HIF1α in nuclei remained dropping from 48 h even though Sirpα was overexpressed (Fig. 7D). To address the issue, the purified mouse CD47 was added to the macrophage culture medium to activate Sirpα. Our data showed that CD47 stimulation reversed the recession of HIF1α in the Sirpα overexpressed macrophages nuclei (Fig. 7E). HIF1α in the macrophage nuclei maintained a relatively high level, even at 168 h post-hypoxic-treatment, compared to the macrophage that received PBS. In addition, without Sirpα overexpression, CD47 did not induce the sustainability of HIF1α in hypoxic macrophages; HIF1α declined from 48 h (sFigure 20). Moreover, the relocation of HIF1α was observed, immunofluorescence indicated that more HIF1α was relocated into the nuclei in the Sirpα overexpressed macrophages that were incubated in hypoxic conditions with the presence of CD47 compared to the macrophages incubated in hypoxia without the presence of CD47 (Fig. 7F and sFigure21). Stabilized HIF1α promoted growth factors such as VEGF and bFGF secretion (sFigure 22) and further facilitated endothelial cell proliferation (sFigure 23), migration (sFigure 24), and tube formation (sFigure 25) [32, 33].

Our results show that the activation of Sirpα promotes stability and relocation of HIF1α in the macrophages incubated in the hypoxia. Relocated HIF1α further initiates the expression of the angiogenesis-related gene, thus accelerating endothelial cell proliferation, migration, and tube formation [34].

**LPS and IL-4 function in a concerted way to alleviate mouse myocardial infarction by mediating Sirp**α.

**Fig. 8:**
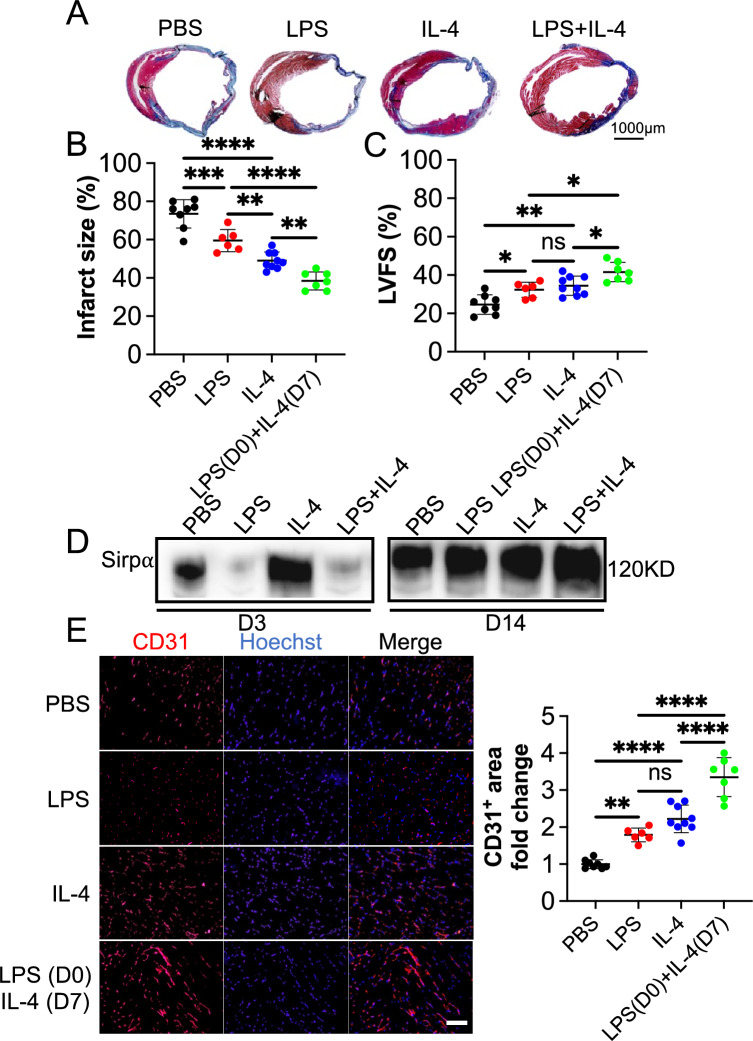
LPS and IL-4 worked in a coordinated way to promote angiogenesis and function recovery in the mouse ischemic heart model. Mouse myocardial infarction (MI) was induced by permanently ligating the left anterior descending (LAD) coronary artery. PBS (PBS, n = 8), LPS at early-stage (LPS, n = 6), IL-4 at late-stage (IL-4, n = 9) or LPS at early-stage plus IL-4 at late-stage (LPS (D0) + IL-4 (D7), n = 7) were tail vein injected, respectively. Four weeks post-surgery, cardiac function was evaluated with echocardiography, and hearts were collected thereafter for morphological examination. **A** and** B** Infarct size was evaluated with the fibrotic area of the left ventricle by using Masson’s trichrome staining. The blue color represents fibrosis (scale bar: 1000 µm). **C** Left ventricular fractional shortening (LVFS) was determined with echocardiography. **D** The level of Sirpα in the infarcted heart was determined by using immunoblotting (n = 4). **E** The sections of the hearts were subjected to immunohistochemistry analysis of CD31 and counterstained with DAPI (scale bar: 100 µm). The CD31^+^ area in the PBS group was set to 1. Data is analyzed using one-way ANOVA followed by Tukey multiple range test and expressed as mean ± SD. *p < 0.05, **p < 0.01, ***p < 0.001, and ****p < 0.0001. ns, non-significant

Our results above demonstrated that LPS and IL-4 promoted angiogenesis in a mouse hindlimb ischemia model and in vitro by modulating the Sirpα level in macrophages. The effect on a mouse MI model was evaluated to further study the proangiogenic effect of Sirpα and the underlying mechanism of ischemic heart disease. Four weeks post ligation of LAD coronary artery, the results from the Masson's staining of the harvested hearts revealed the percentage of LV infarct size in mice that received both LPS at early-stage and IL-4 at late-stage (LPS + IL-4) treatment was smaller (Fig. 8A and 8B). The echocardiography data showed that the rate of left ventricular fractional shortening (LVFS) was higher in mice that received LPS + IL-4 than those that received PBS, LPS, or IL-4 (Fig. 8C). LPS repressed the expression of Sirpα in the macrophages collected from the infarcted heart at early-stage, and repression was mitigated when the mouse administrated IL-4 at the late-stage (Fig. 8D and sFigure 26). Meanwhile, the results of immunohistochemistry analysis showed that the CD31 positive area was elevated in the mouse heart, which received LPS, IL-4, or LPS + IL-4. Similarly, the LPS + IL-4 treatment induced the largest CD31^+^ area in the MI mouse heart (Fig. 8E). These findings suggest that LPS + IL4 treatment significantly improves cardiac function, reduces infarct size, and promote angiogenesis in mouse infarcted heart, compared to the use of LPS or IL-4 alone, administrating LPS at the early-stage combined with IL-4 at the late-stage arouses much better MI resilience, exhibits the coordination of LPS and IL-4. In addition, LPS or IL-4 administrating induced the down and up of Sirpα, indicating the pro-angiogenic effect of LPS or IL-4, at least partially, via modulating Sirpα in macrophages infiltrated in MI mouse heart.

## Discussion

In modern society, ischemic diseases, including ischemic heart disease, leads to the second-highest number of mortalities around the world [35]. Traditionally, promoting angiogenesis is recognized as one of the best therapies for ischemic illnesses and has evolved in the past decades [36]. Recently, the role of the immune system, primarily the innate immune system, in treating ischemic diseases has been revealed and emphasized[37]. However, there is a broad gap in our understanding of how the immune system modulates angiogenesis in ischemic diseases; the system is verified as both facilitating and inhibiting angiogenesis [25, 38]. In our studies, we reported that both pro-inflammatory (M1-like) and anti-inflammatory (M2-like) macrophages promote angiogenesis in mouse ischemic hindlimb and heart. The pro-angiogenic effect was achieved by regulating the Sirpα in macrophages that infiltrated in ischemic tissue, in an exact time-dependent manner.

Macrophages are one of our body's most diverse innate immune cells and participates in nearly every aspect of life[39]. The plasticity of macrophages endows their ability to polarize to different phenotypes to conquer various challenges. Its role as a balance guardian responds to unbalance clues which may cause fatal consequences. Although its role in treating ischemic diseases has been validated, there is much to be elucidated. Anti-inflammatory polarized macrophage is broadly reported to facilitate the resilience of nearly all kinds of ischemic diseases [40–42]. Its opposite counterpart, the pro-inflammatory activated macrophage, traditionally was identified as the ischemic disease deterioration inducer [43–45]. However, a recent report points out that LPS-activated macrophages (pro-inflammatory) promote the alleviation of mouse MI by consuming the apoptotic cells in a highly efficient way [13]. These findings indicate the subtle role of these disparate-polarized macrophages in treating ischemic diseases. Pro-inflammatory polarization favors the clearance of apoptotic debris induced by hypoxia [46]. Anti-inflammatory activation facilitates tissue repair by secreting anti-inflammatory cytokines and growth factors [12, 25]. Our findings confirmed that early-stage injection of LPS promoted pro-inflammatory activation of macrophages which accelerated the cleaning of hypoxia-generated apoptotic debris and led to the turnover of the microenvironment of ischemic tissue from pro-inflammation to pro-tissue repair. IL-4 which induced anti-inflammatory polarization of macrophages enhanced the pro-angiogenic cytokines and growth factors production at the late-stage. More important these findings exhibit a rigid time-dependent behavior; only early-stage administration of LPS and late-stage utilization of IL-4 rouses significant therapeutic effect on ischemic disease. Administrating these treatments at inappropriate times leads to an attenuation of angiogenesis, even promoting the deterioration of ischemic diseases.

Further, the analysis of the underlying mechanisms indicated that Sirpα, a signaling protein reported regulating activation of macrophages, mediated LPS and IL-4 induced therapeutic effects. Sirpα has been reported to regulate macrophage activation through CD47-Siprα axis. Down regulation of Sirpα results in the attenuation of macrophage phagocytosis activity. It has been reported that tumor employs CD47-Sirpα to inactivating macrophage and achieves immune evasion. Hypoxia induces apoptosis; to survive, the cells elevate the level of CD47, which inhibits macrophage activity by ligating Sirpα, to escape from macrophage phagocytosis. CD47-Sirpα axis deactivated macrophages fail to clean apoptotic debris thus boosting the accumulation of inflammation inducers, which cause long-lasting inflammation at ischemic sites. Knockdown of Sirpα at the early-stage revitalizes macrophages thus accelerating the microenvironment turns into a pro-tissue repair scenario at ischemic sites. Moreover, our previous study demonstrated that miR-17 ~ 92 cluster regulated by c-Myc, post-transcriptional regulates Sirpα expression in macrophages [15]. Stat3 phosphorylation is reported to enhance c-Myc expression and activation [47]. In our study, by knocking down, Stat3 in the LPS-treated macrophages, the level of Sirpα has augmented duo to the deactivation of c-Myc-induced reduction of miR-17 ~ 92 cluster. Stat3 knocked down macrophages that were incapable of cleaning apoptotic debris and resulted in the crippling of LPS-induced therapeutic effects.

In late-stage, it is necessary to promote angiogenesis and re-establish blood flow. Anti-inflammatory polarized macrophages have demonstrated its importance in ischemic disease treatments. HIF1α, a potent hypoxia-inducible transcription factor, is considered as the master of response to hypoxia. Although, HIF1α level increases swiftly during the initiation of hypoxia, due to the negative feedback loop [48], its level drops sharply even though continued hypoxia. Maintaining HIF1α level during the late-stage of ischemic diseases benefits to the therapy of the disease. Our study revealed that although overexpressing Sirpα alone was not enough to preserve the level of HIF1α, activating Sirpα with CD47 stabilized HIF1α expression. More interestingly, the presence of CD47 promotes more HIF1α relocated to the nucleus in the Sirpα overexpressed macrophage. The stabilization and relocation of HIF1α may be partly because of recruiting SHP-2 by CD47 ligated Sirpα, and it has been reported that SHP-2 promotes the activation of HIF1α [49]. Activated HIF1α initiates angiogenesis-associated gene transcription and contributes to the shifting of macrophage secretome from pro-inflammation to anti-inflammation and pro-angiogenesis, which is essential for successful tissue repair [50].

## Conclusion

Our results reveal that Sirpα contributes to macrophage polarization-induced alleviation of ischemic hindlimb and heart. Downregulation of Sirpα in macrophages at the early-stage of the ischemic disease could promote apoptotic debris clearance. On the other side, at the late-stage, upregulation of Sirpα in the macrophage induces relocation of HIF1α and leads to the successful angiogenesis. The phosphorylation of Stat3 and Stat6 mediates the increase or decrease of Sirpα in the macrophage. Moreover, the presence or not of CD47 plays a crucial role in Sirpα induced angiogenic effect. This study emphasizes the importance of time-point in ischemic disease treatment and introduces Sirpα as a new therapeutic target, and expands our understanding of the underlying mechanism of macrophage polarization facilitated ischemic disease recovery. The effect of the Sirpα on the regulation of macrophage activation may also imply their broader application for other macrophage activation-related disease treatments.

## Supplementary Information

Supplementary Material 1

## Data Availability

The data that support the findings of this study are available from the corresponding author upon reasonable request.
