# Supplementary material for: Signal regulatory protein α dynamically mediates macrophage polarization facilitated alleviation of ischemic diseases
Source: Cell Biosci. 2024 Dec 20;14:150. doi: 10.1186/s13578-024-01325-2 (PMC11662717; doi:10.1186/s13578-024-01325-2)
Supplement: Supplementary file 1 — Supplementary Material 1 [file 13578_2024_1325_MOESM1_ESM.pdf]

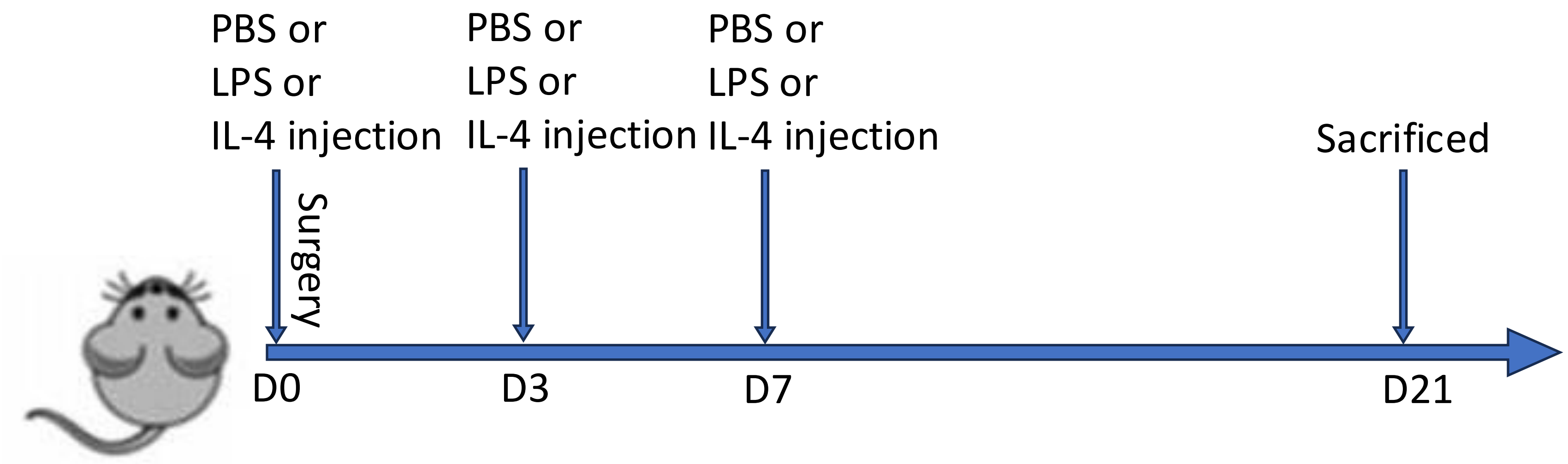

sFigure 1. A scheme for the experimental setup of Figure 1.

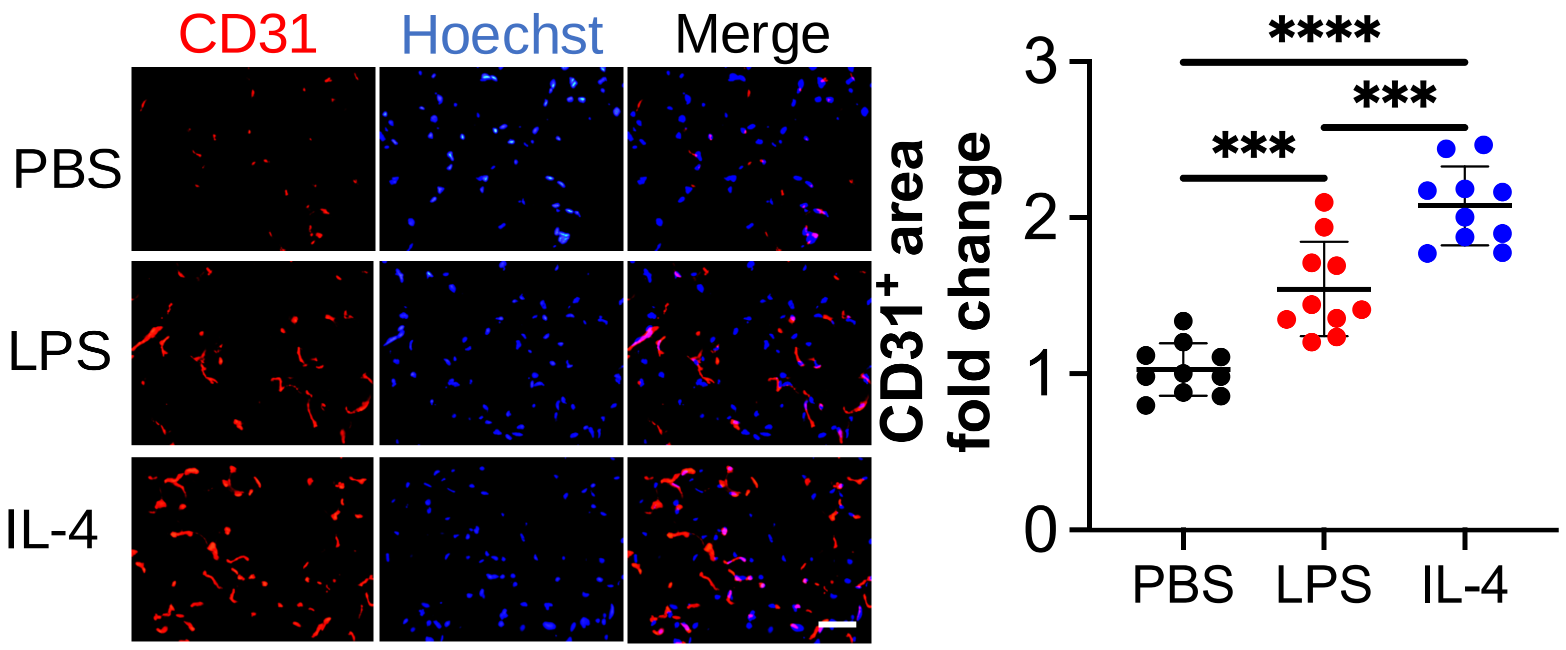

sFigure 2. LPS and IL-4 promoted angiogenesis in a mouse HLI model. After left artery ligation, the mice were received PBS, LPS or IL-4 intramuscularly injection (n=10). The mice were euthanized at 21 days post-surgery. The sections of the gastrocnemius muscle from the ligated side were subjected to immunohistochemistry analysis for CD31 and counterstained with Hoechst 33342 (scale bar: 100 μm). Quantification of the $CD31^+$ area. The $CD31^+$ area on the slide from the mouse administered with PBS was set to 1. Data is analyzed using one-way ANOVA followed by Tukey multiple range test and expressed as mean ± SD , ***p<0.001 and ****p<0.0001.

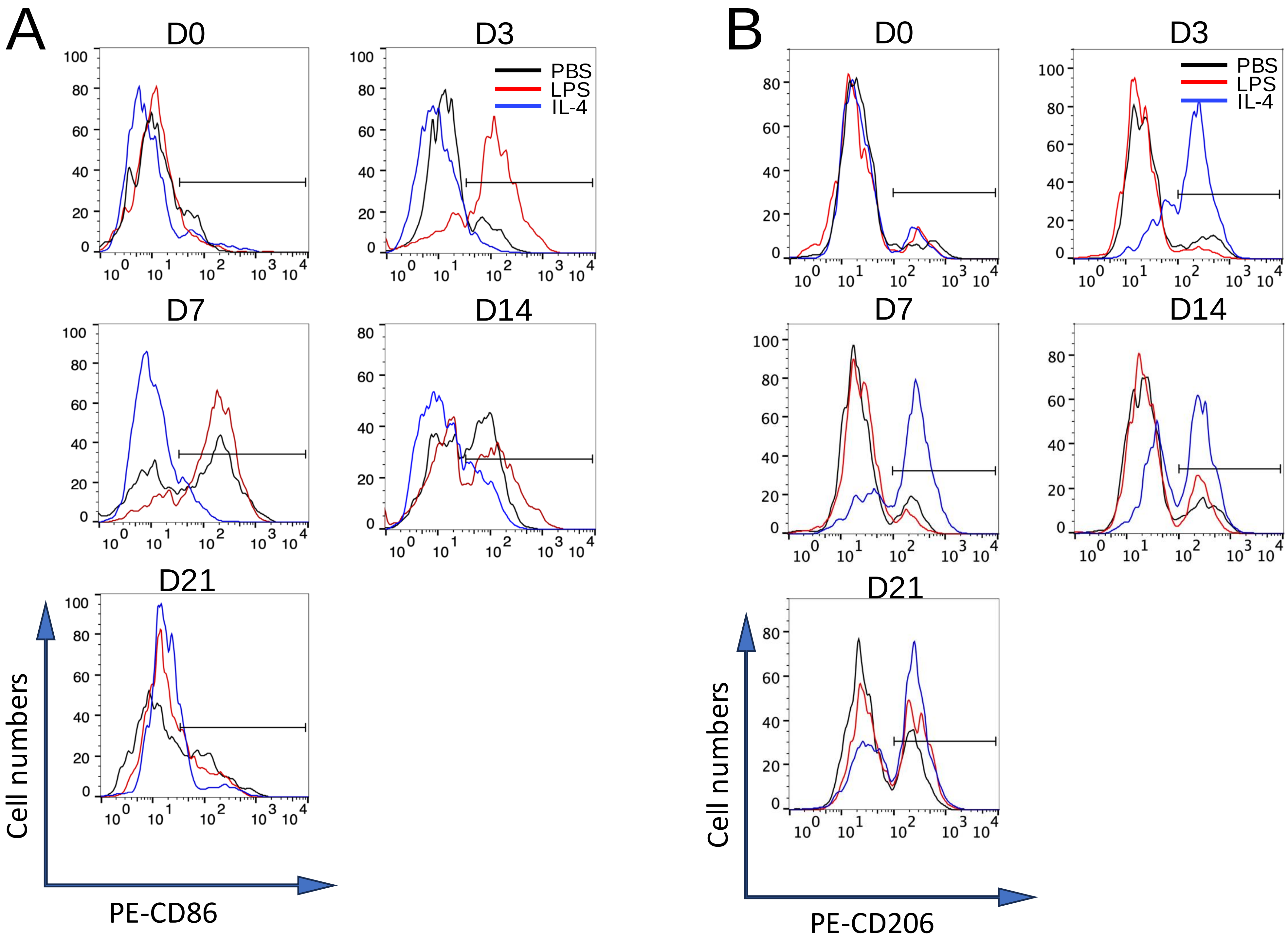

sFigure 3. LPS and IL-4 induced macrophage polarization in a mouse HLI model After left artery ligation, the mice received PBS, LPS and IL-4 intramuscular injection on day 0, 3 and 7 post-surgery (n=5). Macrophage were isolated from adductor muscles and the surface markers, CD86 (pro-inflammatory polarization marker, A), and CD206 (anti-inflammatory polarization marker, B) were checked using flow cytometry. Total 5000 cells were gated and analyzed.

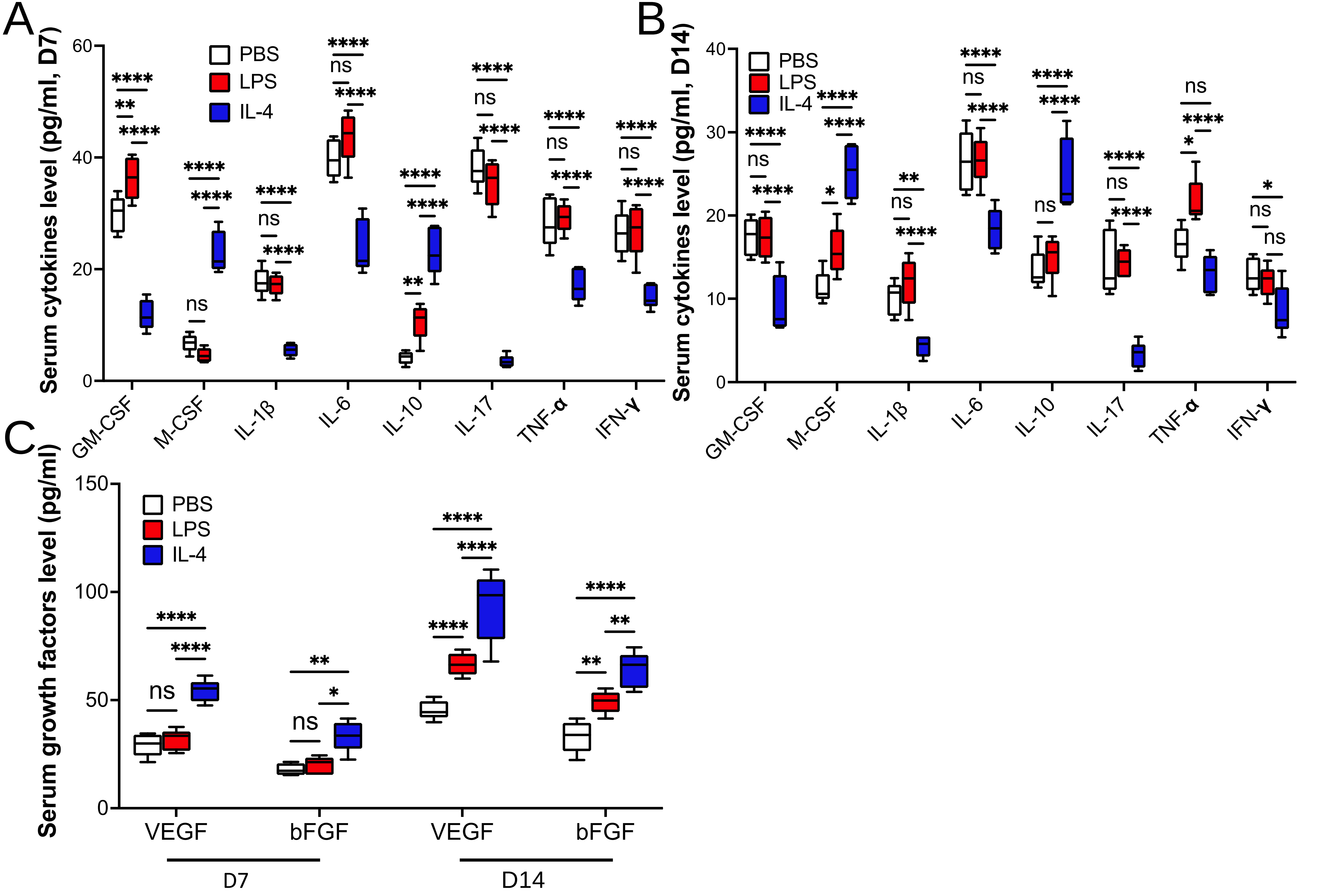

sFigure 4. Cytokines and growth factors in the serum from the ischemic mice were received PBS, LPS and IL-4 intramuscularly injection (n=10). The cytokines in the serum on D7 (A) and D14 (B) post-surgery and growth factors (C) in the serum were measured using ELISA. Data is analyzed using one-way ANOVA followed by Tukey multiple range test and expressed as mean ± SD , **p*<0.05, ***p*<0.01, ****p*<0.001 and *****p*<0.0001. ns, non-significant .

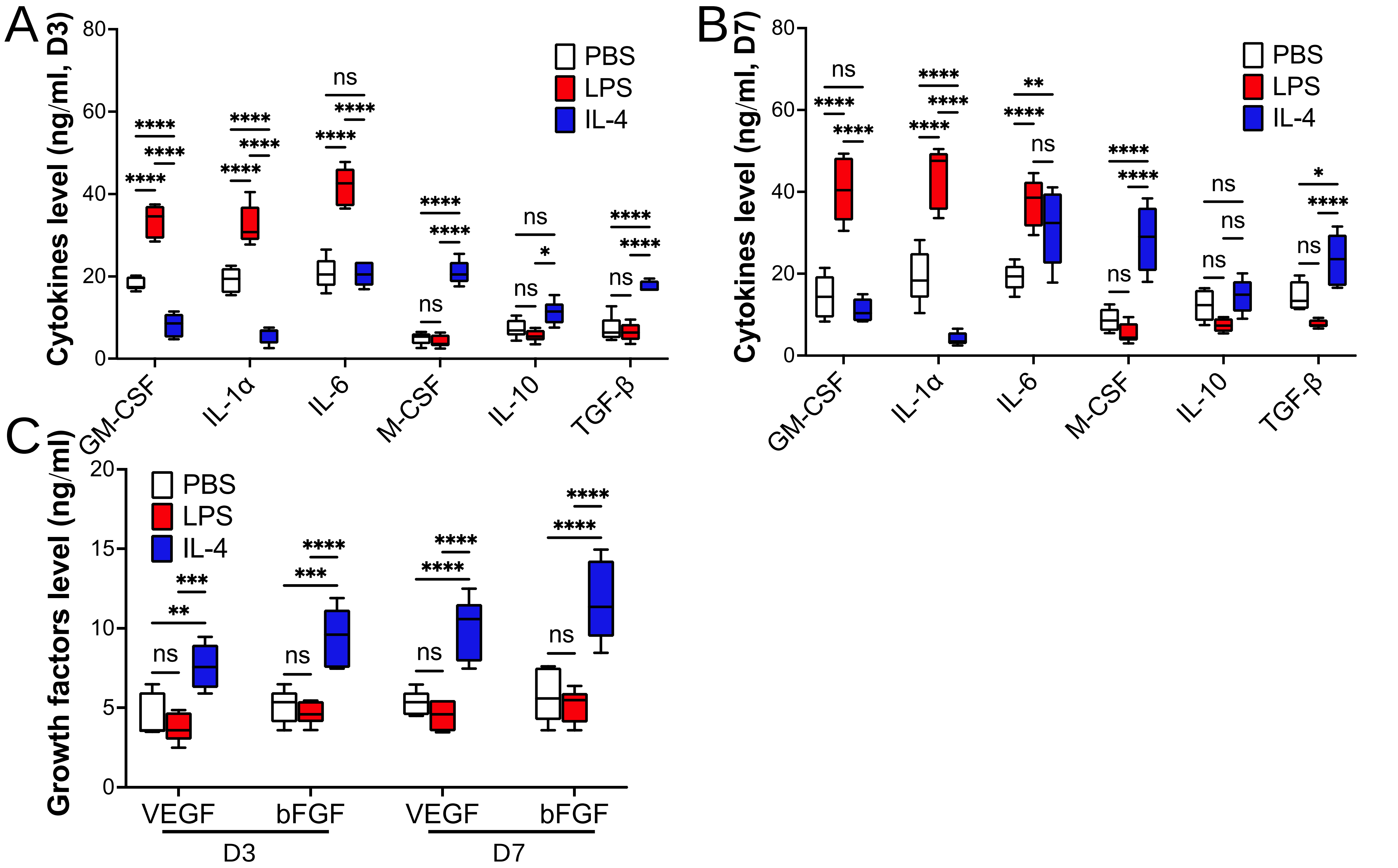

sFigure 5. The factors secreted by macrophages collected from adductor muscles of mice on the indicated days post-surgery, were measured using ELISA. **A**, The cytokines secreted by the macrophages, 3 days post-surgery. **B**, the cytokines secreted by the macrophages, 7 days post-surgery. **C**, Growth factors secreted by the macrophages. Data is analyzed using one-way ANOVA followed by Tukey multiple range test and expressed as mean ± SD of n=5, unless specified. *p<0.05, **p<0.01, ***p<0.001 and ****p<0.0001. ns, non-significant .

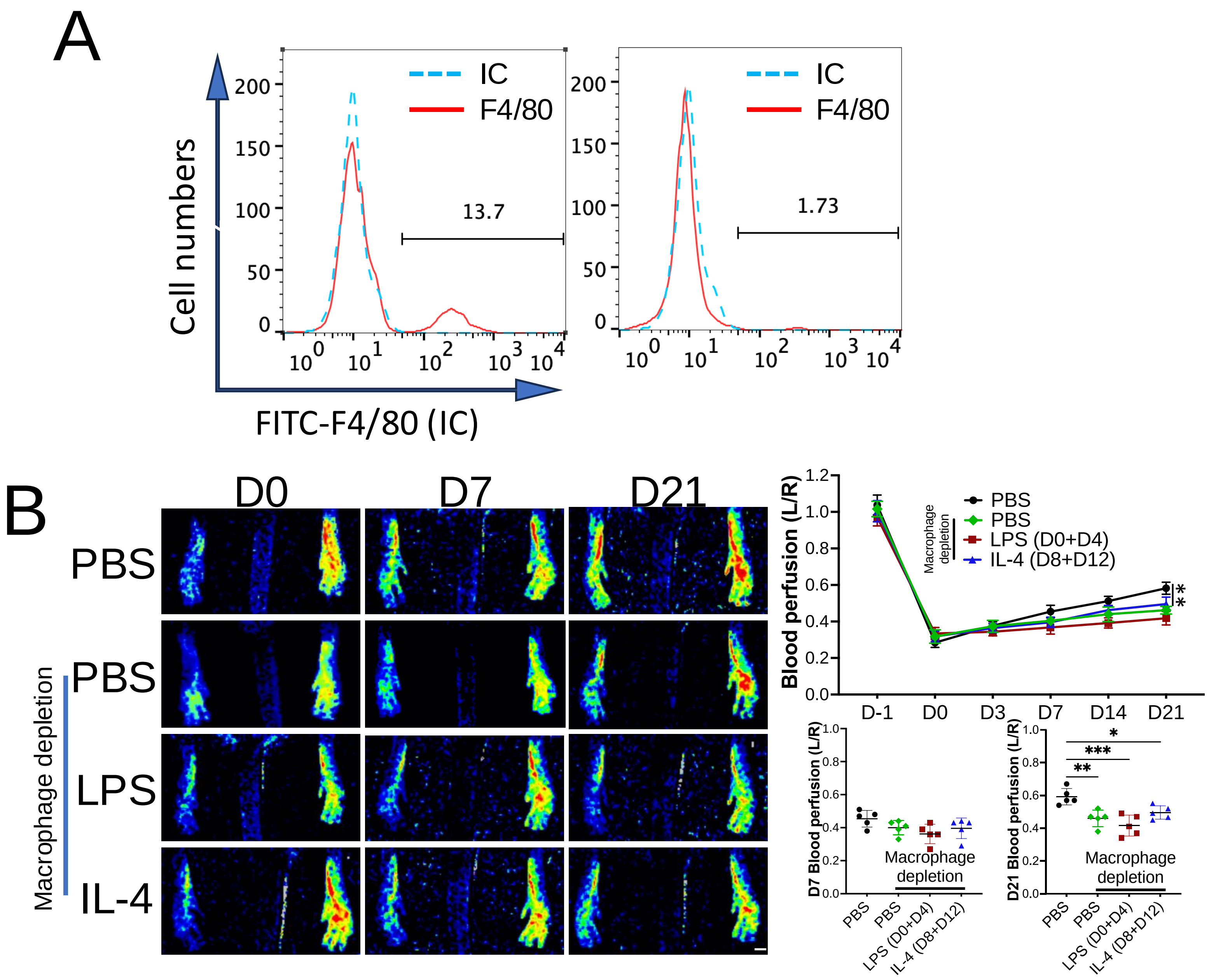

sFigure 6. Macrophage depletion attenuated pro-angiogenic effect of LPS or IL-4 in ischemic mice. After left artery ligation, the mice (n=5) that pre-treated with the clodronate-liposomes to deplete pan macrophage, were received PBS, LPS and IL-4 intramuscularly injection at various time. WT mice received PBS were used as a control. **A**, The percentage of the macrophage in spleen of the mice 24h post clodronate-liposomes injection. Total 5000 cells were analyzed. **B,** Laser speckle images showing the relative level of blood perfusion in the hind paws on the indicated days and the quantitative analyses of the laser speckle images showing the left/right ratio of plantar blood perfusion (D7 and D21, scale bar: 1000 µm). Data is analyzed using one-way ANOVA followed by Tukey multiple range test and expressed as mean ± SD of n=5, unless specified. IC, isotype control, **p*<0.05, ***p*<0.01 and ****p*<0.001.

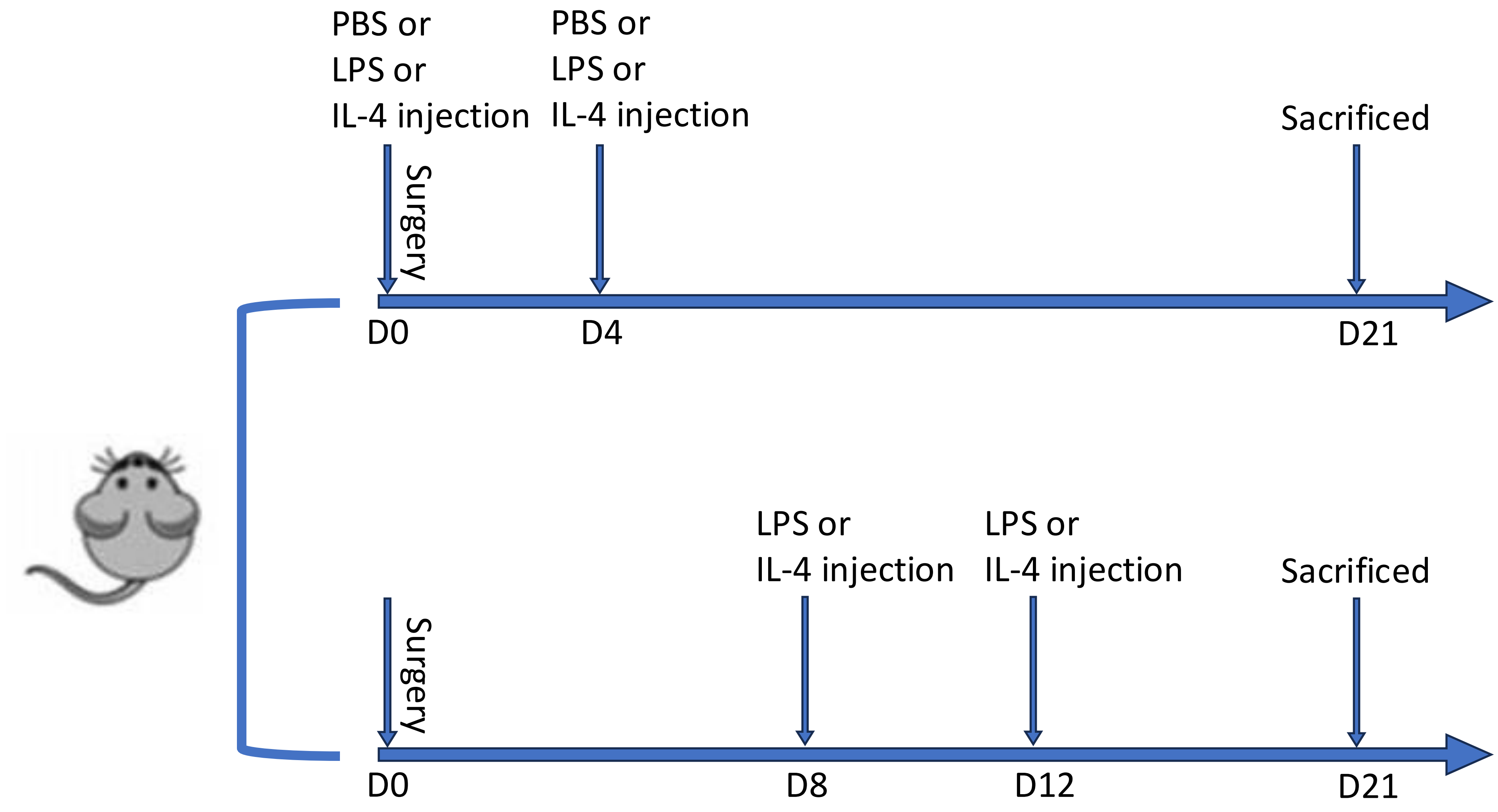

sFigure 7. A scheme for the experimental setup of Figure 2.

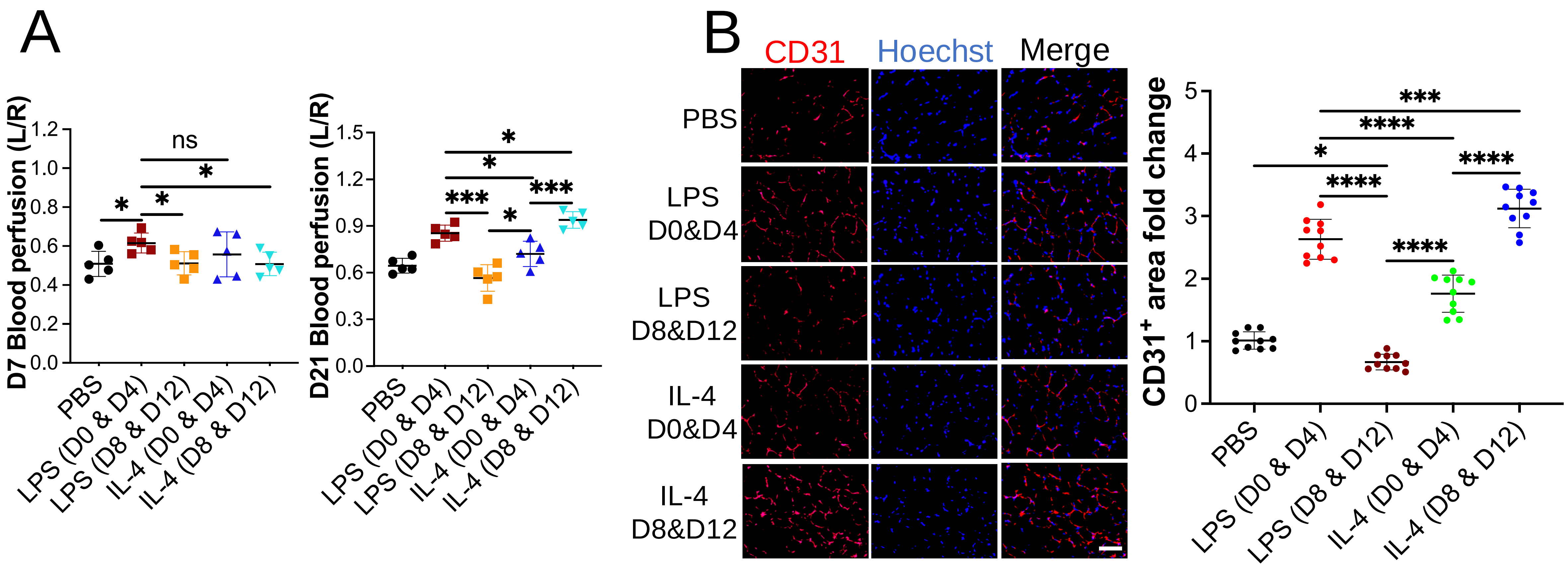

sFigure 8. LPS and IL-4 promoted angiogenesis in mouse hindlimb ischemia in a time-dependent manner. After left artery ligation, the mice were received PBS, LPS and IL-4 intramuscularly injection at various time. **A**, Quantitative analyses of the laser speckle images showing the left/right ratio of plantar blood perfusion (D7 and D21, n=5). **B**, The mice were euthanized 21 days post-surgery. The sections of the gastrocnemius muscle from the ligated side were subjected to immunohistochemistry analysis for CD31 and counterstained with Hoechst 33342 (n=10, scale bar: 100 µm). Quantification of the $CD31^+$ area. The $CD31^+$ area on the slide from the mouse administered with PBS was set to 1. PBS, LPS D0 & D4 or IL-4 D0 & D4: the mice received PBS, LPS or IL-4 intramuscular injection on day 0 and day 4 post-surgery. LPS D8 & D12 or IL-4 D8 & D12: the mice received LPS or IL-4 intramuscular injection on day 8 and day 12 post-surgery. Data is analyzed using one-way ANOVA followed by Tukey multiple range test and expressed as mean ± SD , *p<0.05, ***p<0.001 and ****p<0.0001.

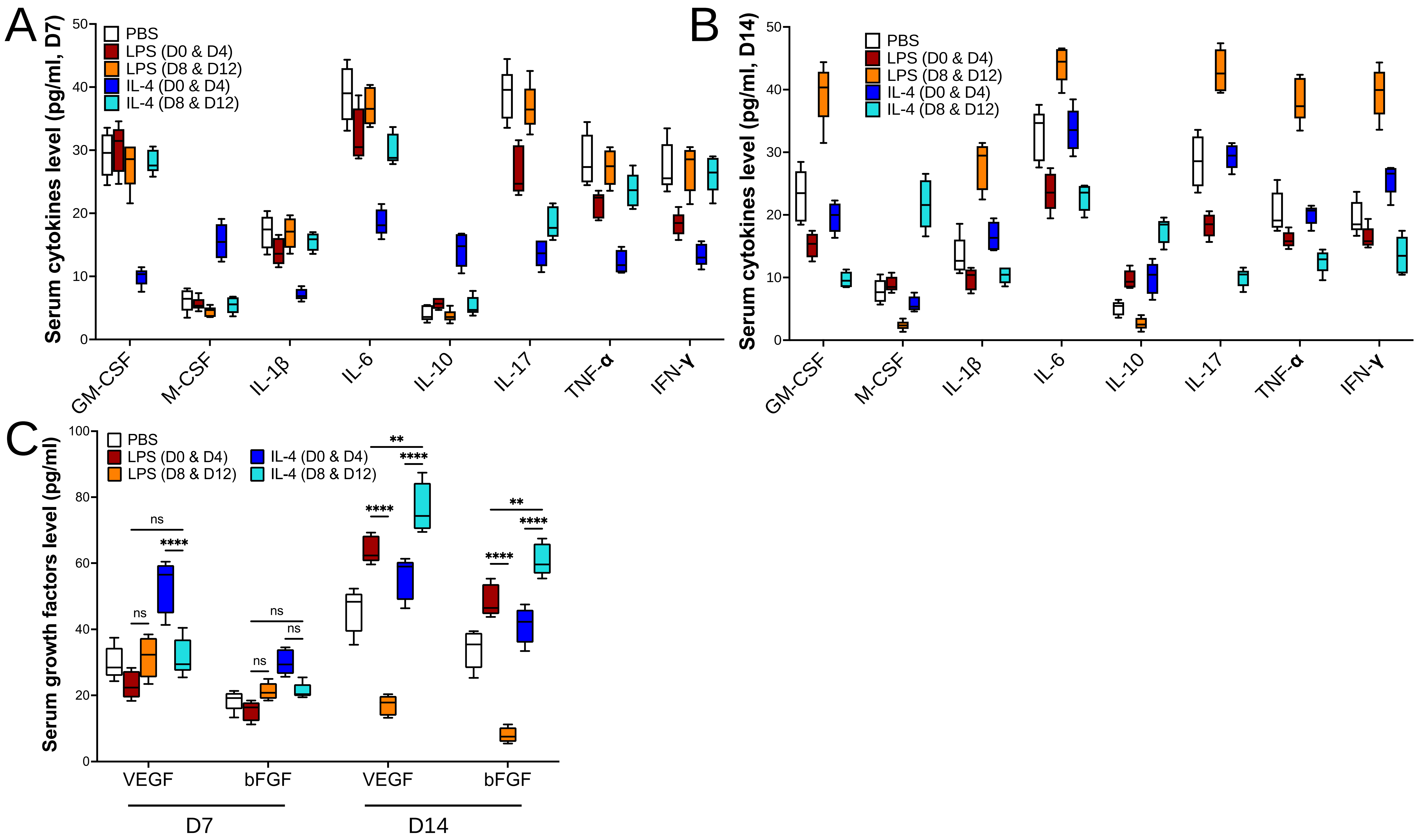

sFigure 9. Cytokines and growth factors in the serum from the mice were received PBS, LPS and IL-4 intramuscularly injection (n=5) at various time, were measured using ELISA. **A and B**, The cytokines in the serum on D7 (A) and D14 (B) post-surgery. **C**, Growth factors level in the serum. PBS, LPS D0 & D4 or IL-4 D0 & D4: the mice received PBS, LPS or IL-4 intramuscular injection on day 0 and day 4 post-surgery. LPS D8 & D12 or IL-4 D8 & D12: the mice received LPS or IL-4 intramuscular injection on day 8 and day 12 post-surgery. Data is analyzed using one-way ANOVA followed by Tukey multiple range test and expressed as mean ± SD , ****p*<0.01, *****p*<0.001 and ******p*<0.0001. ns, non-significant.

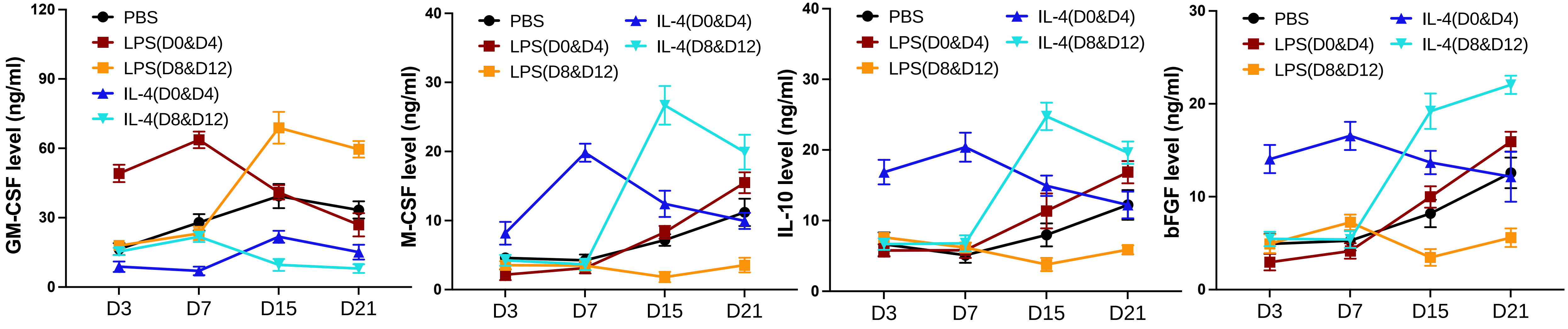

sFigure 10. Cytokines and growth factor secreted by macrophage collected from the hindlimb ischemic model mice that were received PBS, LPS and IL-4 intramuscularly injection (n=5) at various time, were measured using ELISA. . PBS, LPS D0 & D4 or IL-4 D0 & D4: the mice received PBS, LPS or IL-4 intramuscular injection on day 0 and day 4 post-surgery. LPS D8 & D12 or IL-4 D8 & D12: the mice received LPS or IL-4 intramuscular injection on day 8 and day 12 post-surgery. Data is analyzed using one-way ANOVA followed by Tukey multiple range test and expressed as mean ± SD .

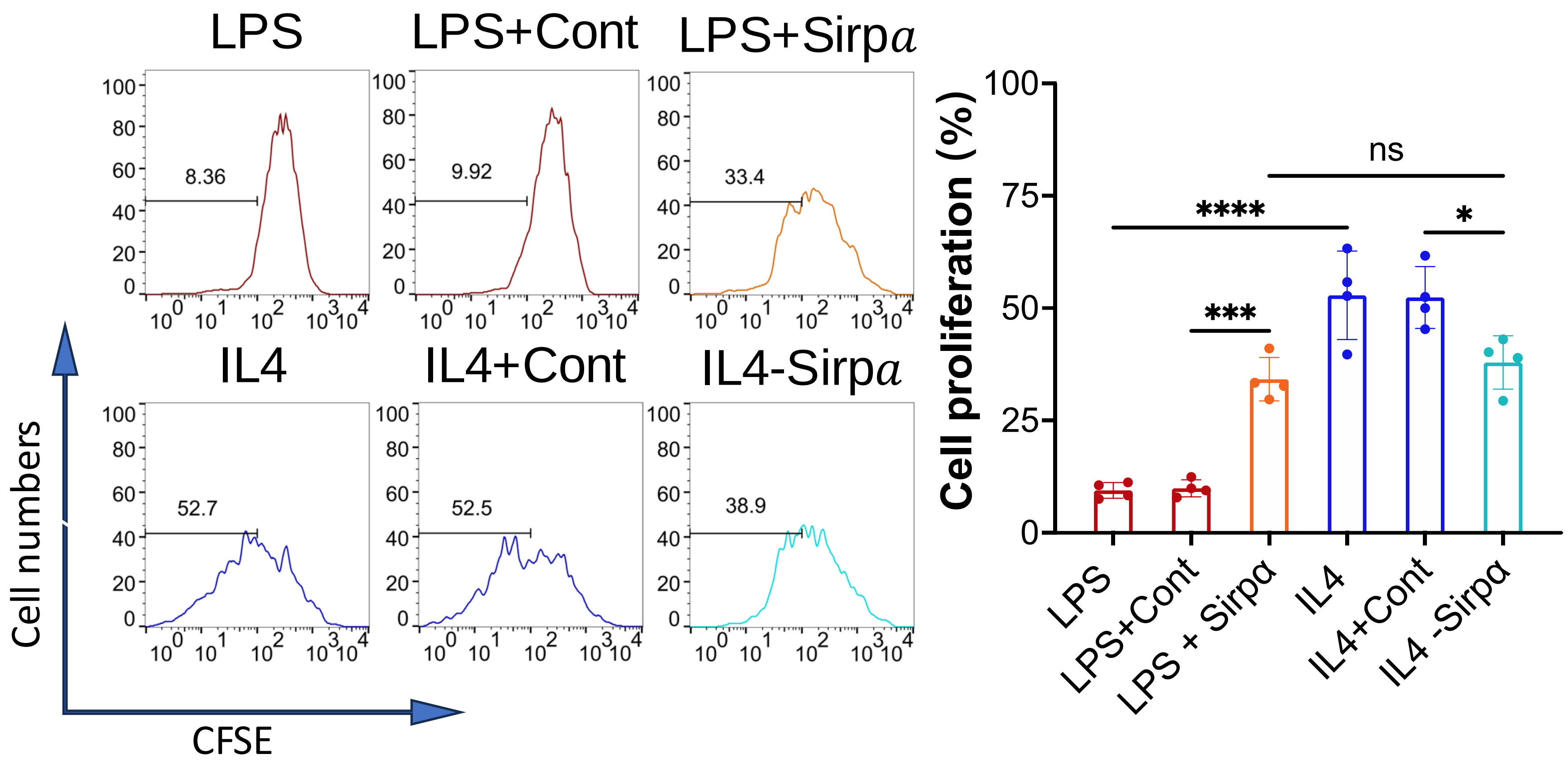

sFigure 11. CMVECs were treated with various conditioned medium (CdM) from macrophages. Cell proliferation assay of the CMVECs were performed. LPS: conditioned medium from LPS treated macrophage, IL4: conditioned medium from IL-4 treated macrophage, LPS+Cont or IL4+Cont: conditioned medium from LPS or IL-4 treated Lentivirus control transfected macrophage, LPS+Sirpα: conditioned medium from LPS treated Sirpα overexpressed macrophage, IL4- Sirpα: conditioned medium from IL-4 treated Sirpα knockdown macrophage. Data is analyzed using one-way ANOVA followed by Tukey multiple range test and expressed as mean ± SD , ***p*<0.01, ****p*<0.001 and *****p*<0.0001. ns, non-significant. Total 5000 cells were analyzed.

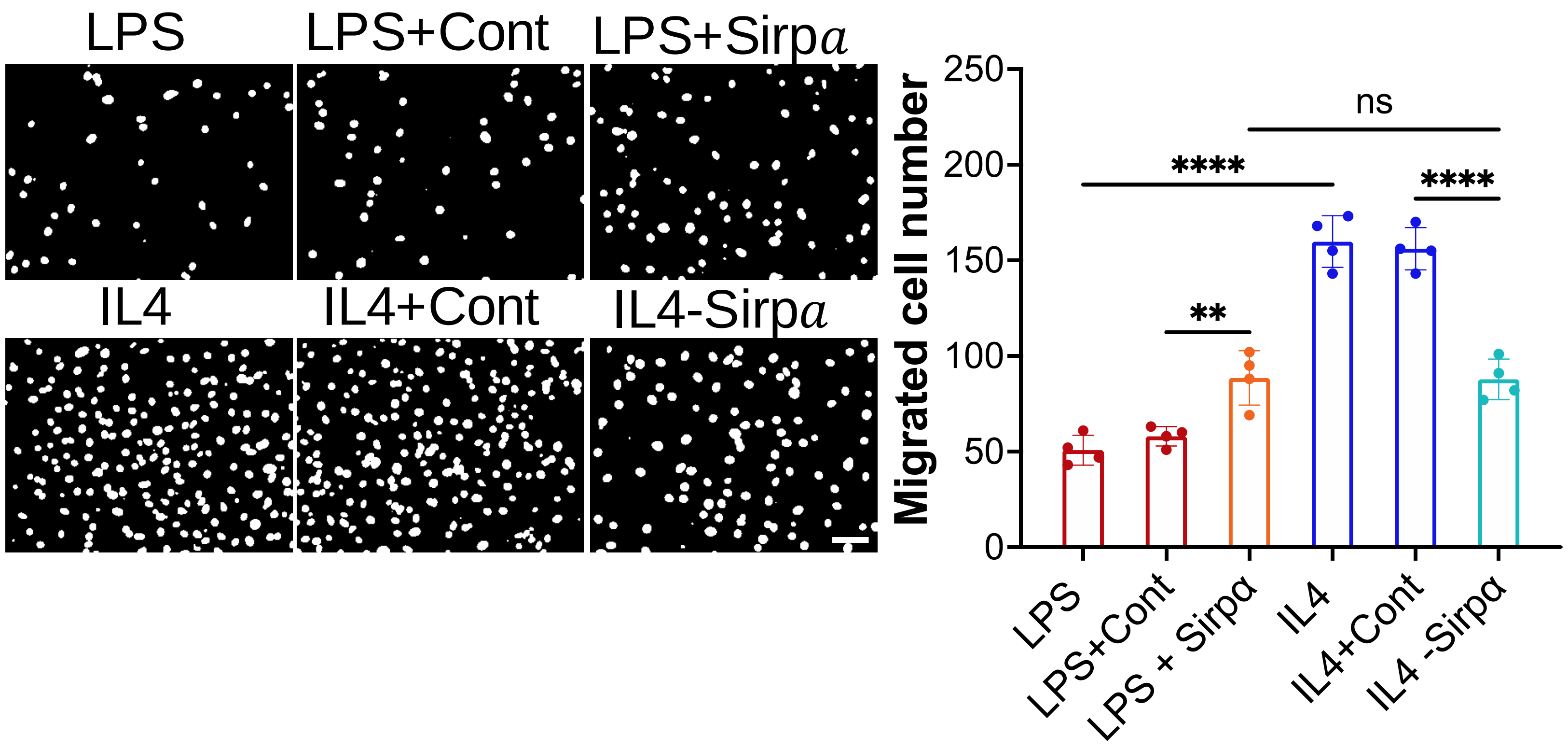

sFigure 12. Cell migration assay of the CMVECs that incubated with the various conditioned medium from the macrophages, were performed, scale bar: 100 µm. Data is analyzed using one-way ANOVA followed by Tukey multiple range test and expressed as mean ± SD , **p<0.01 and ****p<0.0001. ns, non-significant.

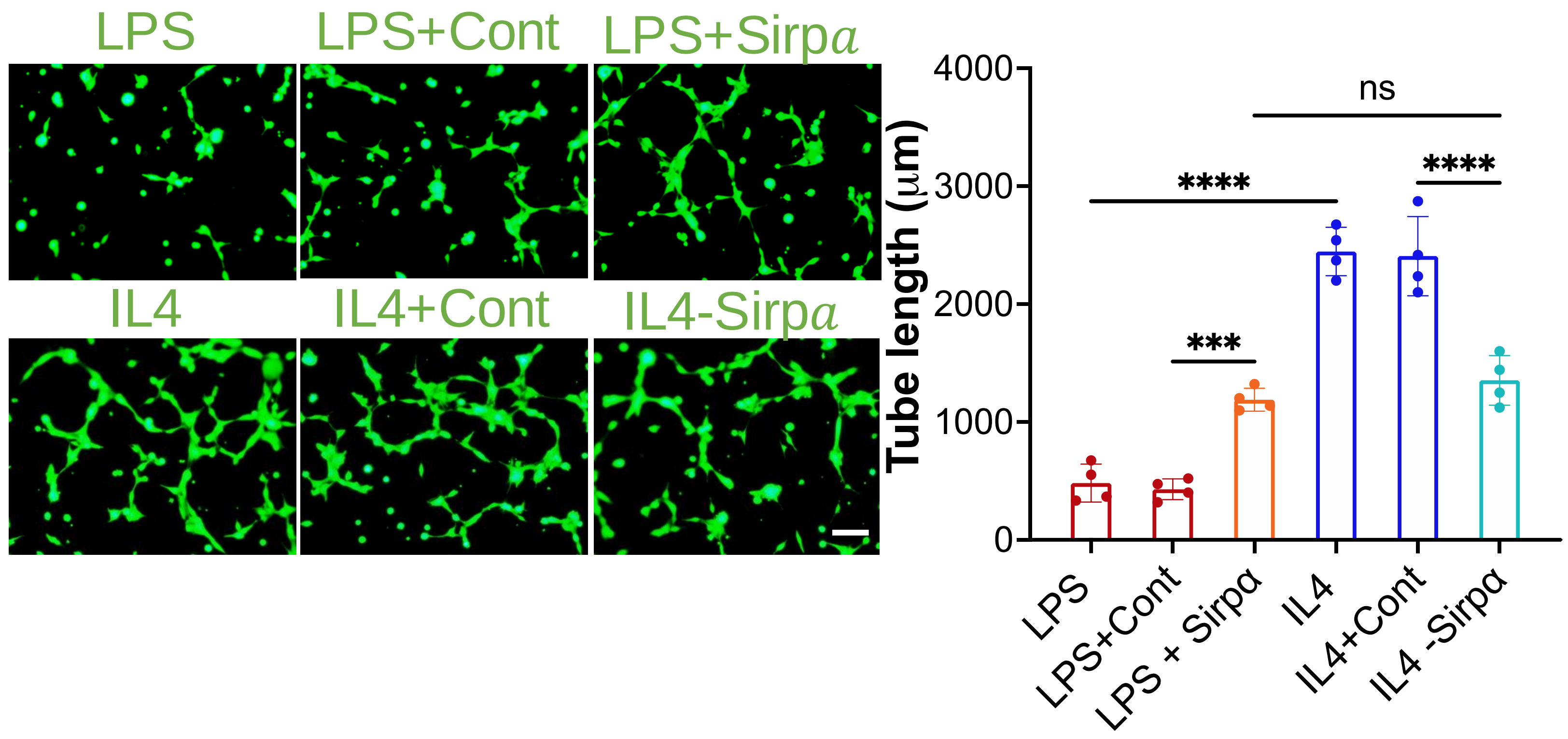

sFigure 13. Tube formation of the CMVECs that incubated with the various conditioned medium from the macrophages, were performed, scale bar: 50 µm. Data is analyzed using one-way ANOVA followed by Tukey multiple range test and expressed as mean ± SD , ***p<0.001 and ****p<0.0001. ns, non-significant.

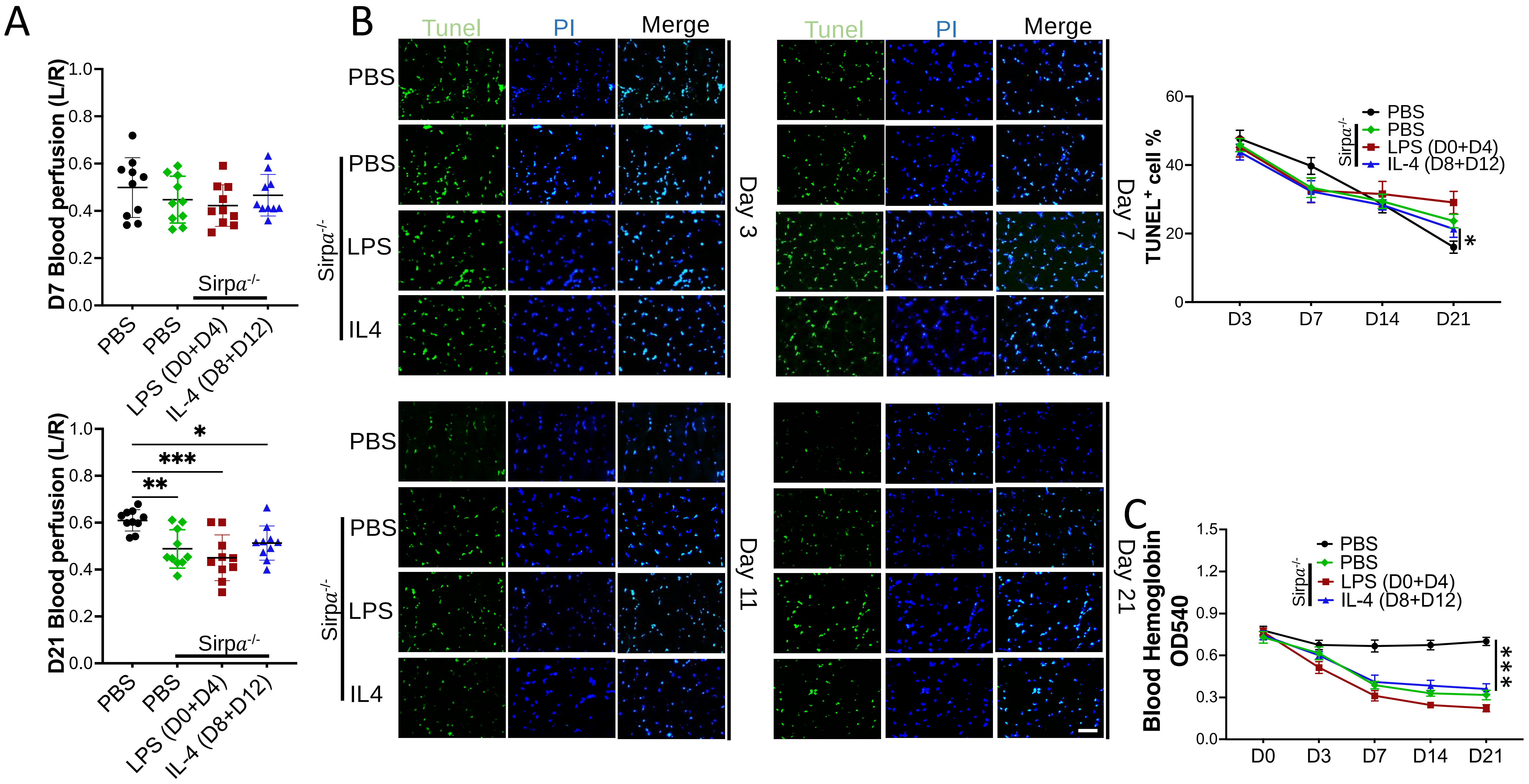

sFigure 14. Sirpα knockout (Sirp$\alpha^{-/-}$) attenuated LPS and IL-4 promoted angiogenesis in a mouse HLI model. After left artery ligation, the mice were received PBS (WT), PBS, LPS and IL-4 (Sirpα knockout) intramuscularly injection on indicated day (n=10). **A**, Quantitative analyses of the laser speckle images showing the left/right ratio of plantar blood perfusion (D7 and D21). **B,** The percentages of dead cells in gastrocnemius muscles on D3, D7, D14, and D21 post-surgery (scale bar: 100 µm). **C,** Blood hemoglobin levels. Data is analyzed using one-way ANOVA followed by Tukey multiple range test and expressed as mean ± SD , **p*<0.05, ***p*<0.01 and ****p*<0.001.

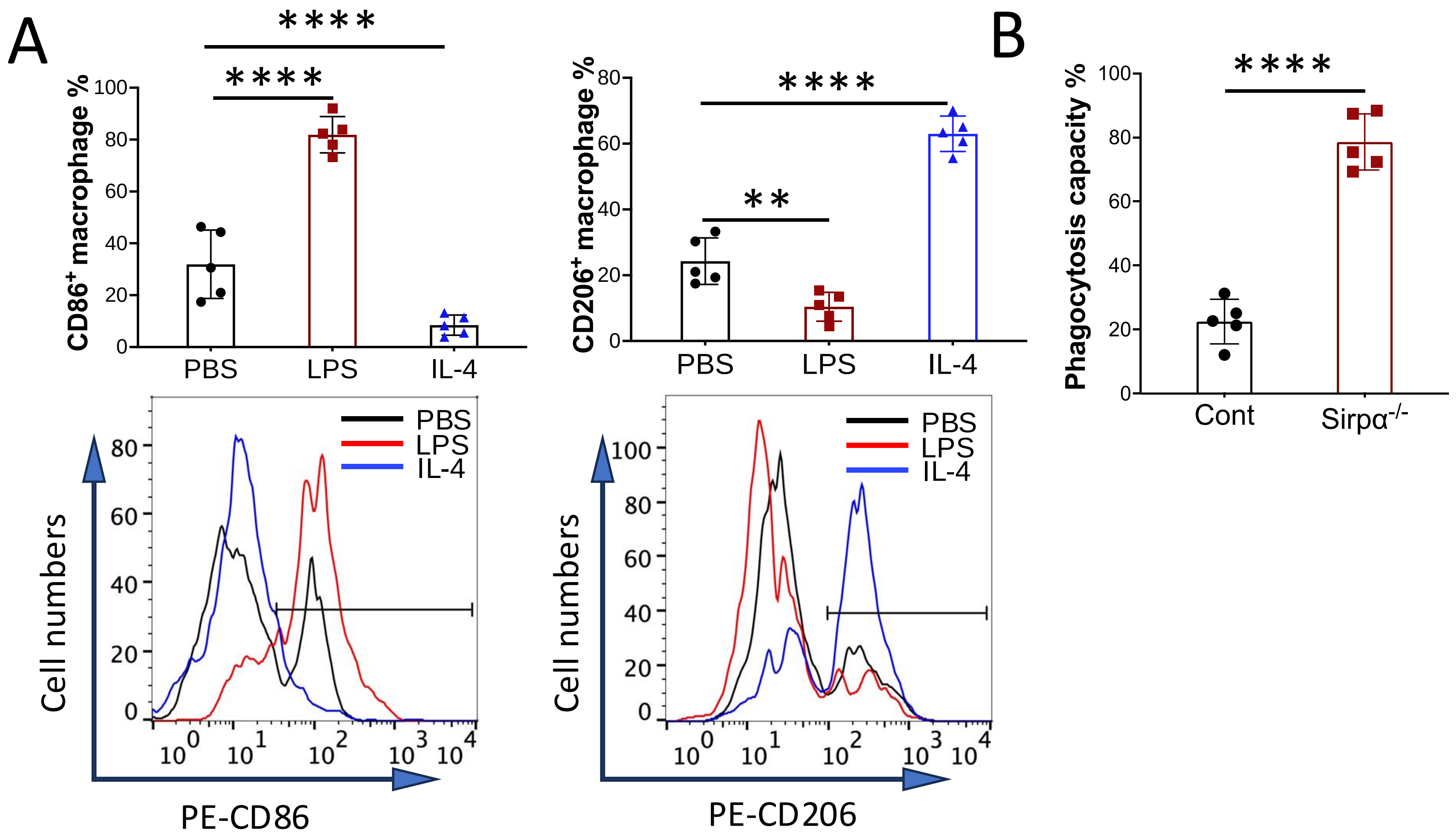

sFigure 15. Sirpα knockout does not affect macrophage polarization, but upregulating the phagocytosis of macrophage. The macrophages were collected from Sirp$\alpha^{-/-}$ mice, and treated with PBS, LPS or IL-4 for 48h, respectively. **A** , The macrophage surface marker CD86 (pro-inflammatory polarization marker) and CD206 (anti-inflammatory polarization marker) were tested by flow cytometry. Total 5000 cells were gated and analyzed. **B**, The phagocytosis of pHrodo Red-labeled apoptotic MCMVEC cells by the macrophages collected from WT (Cont) or Sirp$^{-/-}$ mice (Sirp$^{-/-}$). Data is analyzed using one-way ANOVA followed by Tukey multiple range test and expressed as mean ± SD , **p<0.01 and ****p<0.0001.

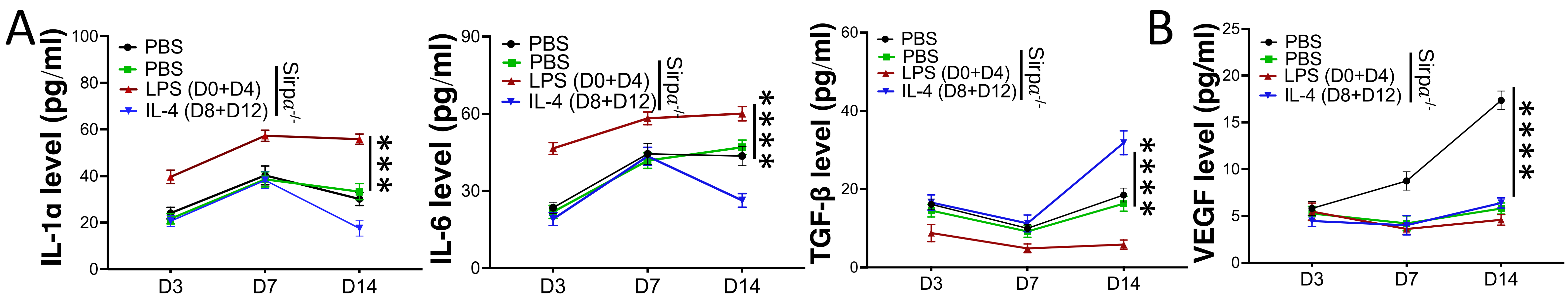

sFigure 16. Cytokines and growth factors in the serum from the hindlimb ischemia model of Sirp$\alpha^{-/-}$ mice received PBS, LPS and IL-4 intramuscularly injection (n=5) on indicated time, were measured using ELISA, WT mice received PBS were used as a control. **A**, The cytokines in the serum. **B**, Growth factor VEGF level. Data is analyzed using one-way ANOVA followed by Tukey multiple range test and expressed as mean ± SD , ****p*<0.001 and *****p*<0.0001.

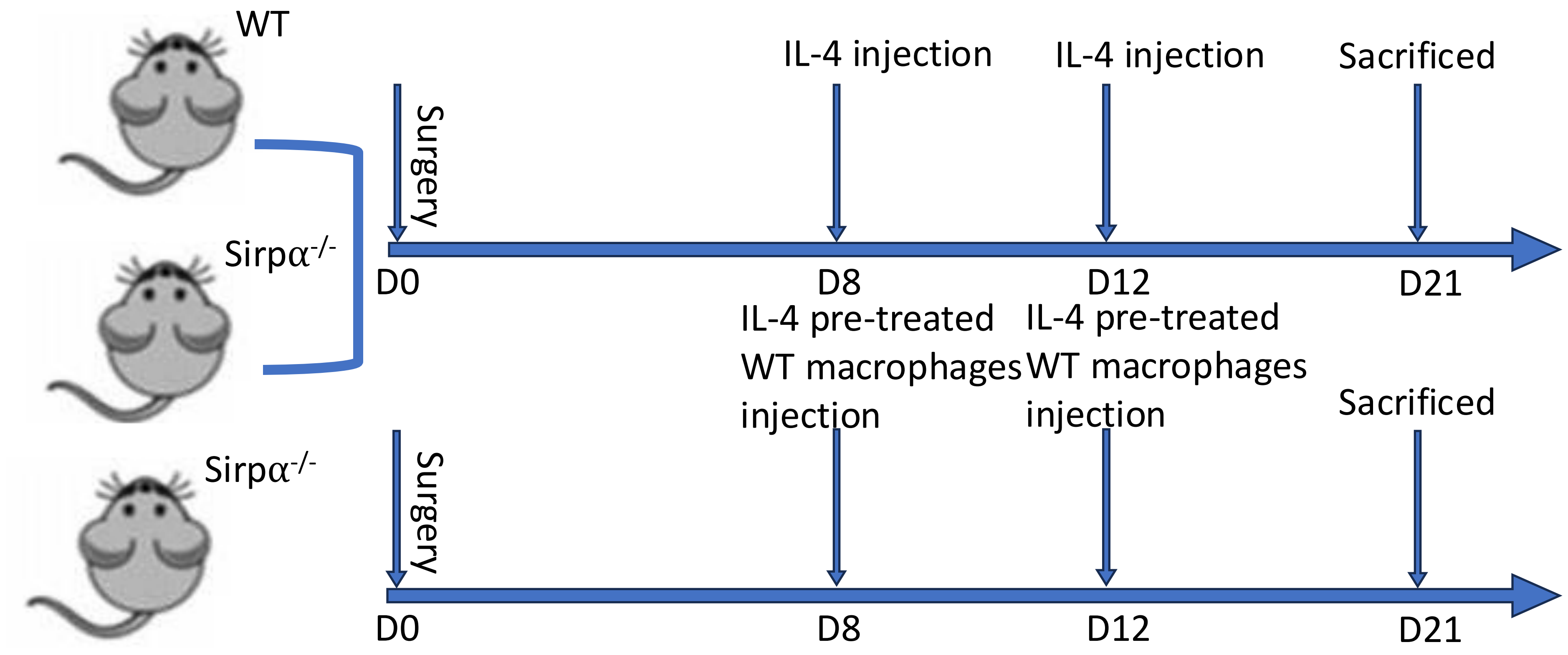

sFigure 17. A scheme for the experimental setup of Figure 4C and 4D.

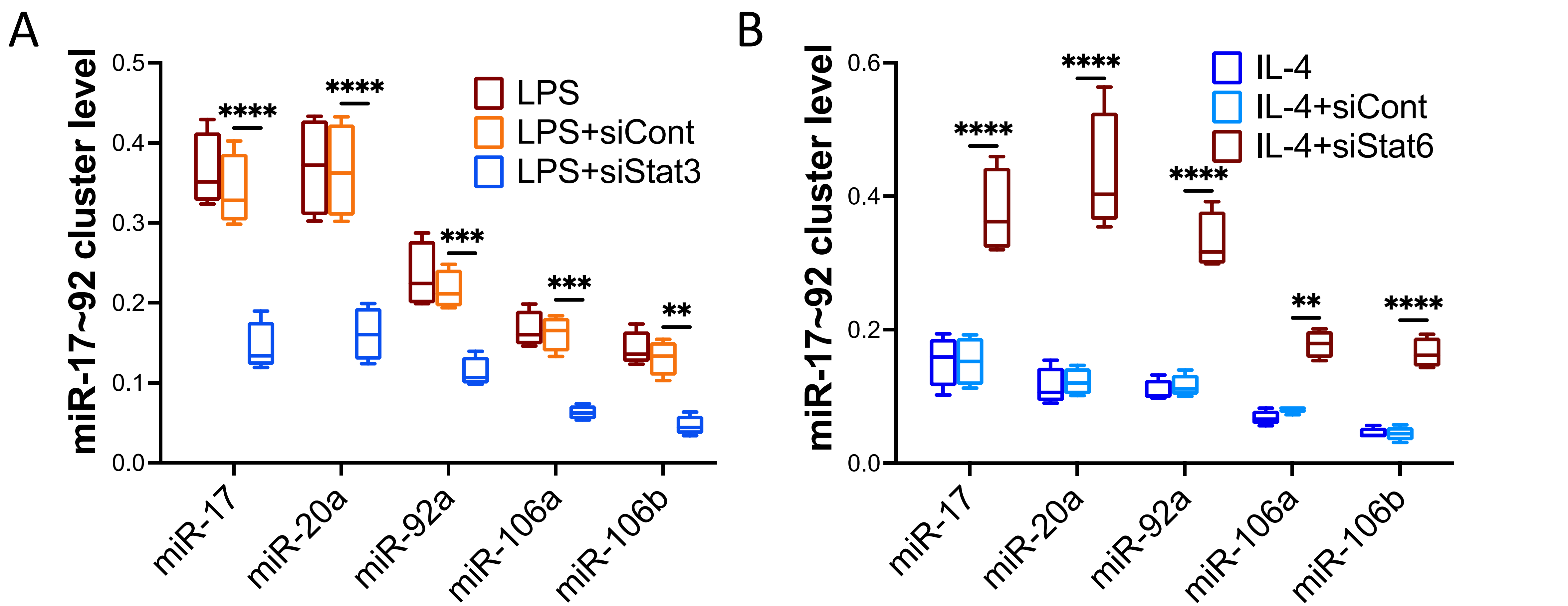

sFigure 18. Macrophages isolated from adductor muscles of WT mice were pre-treated with LPS or IL-4. Lentivirus was used to knock down Stat3 (siStat3) or Stat6 (siStat6) in the macrophage, lentivirus control (siCont) was used as a control. **A and B**, The levels of miR17~92 cluster in the macrophages that received LPS (A) or IL-4 (B), were determined by RT-qPCR (n=4). LPS: the macrophages were treated with LPS, IL-4: the macrophages were treated with IL-4, LPS+siCont or IL-4+siCont: lentivirus control transfected macrophages were treated with LPS or IL-4, LPS+siStat3: Stat3 downregulated macrophages were treated with LPS, IL-4+siStat6: Stat6 downregulated macrophages were treated with IL-4. Data is analyzed using one-way ANOVA followed by Tukey multiple range test and expressed as mean ± SD , ***p*<0.01, ****p*<0.001 and *****p*<0.0001.

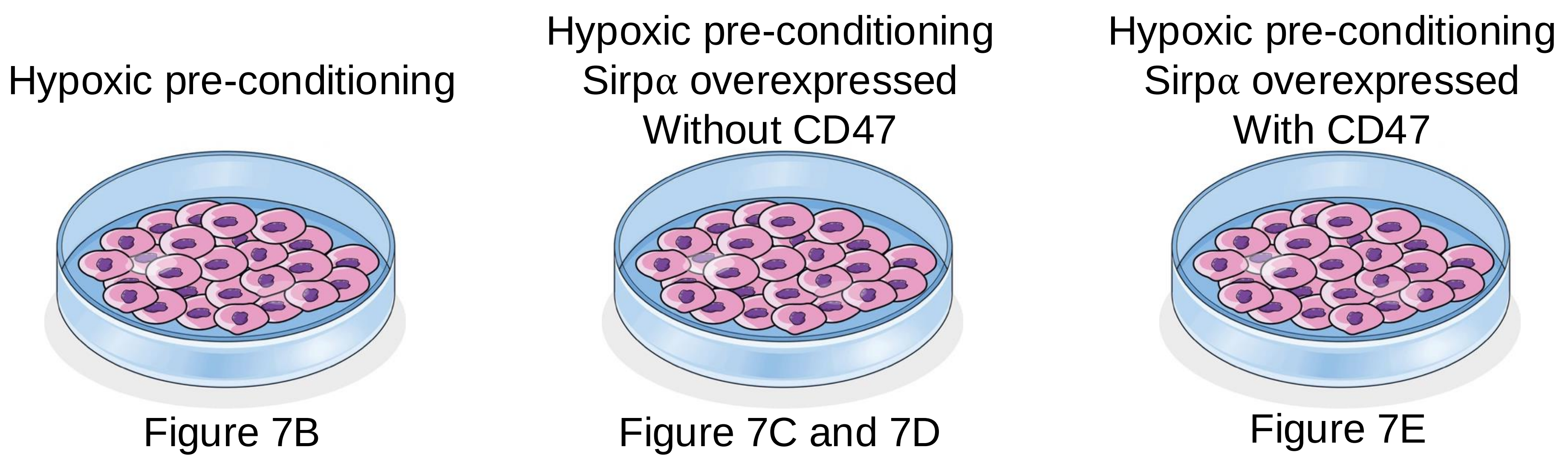

sFigure 19. A scheme for the experimental setup of Figure 7

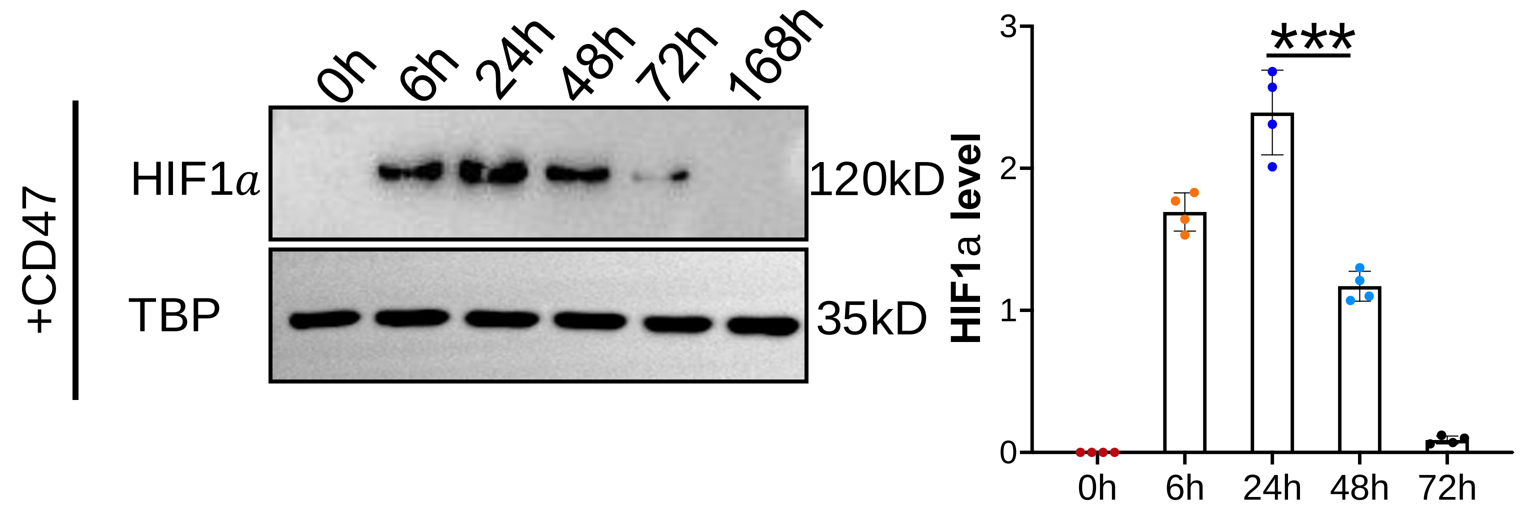

sFigure 20. The macrophages collected from adductor muscles of WT mice were pre-treated with 30ng/ml IL-4 for 48h and then incubated under hypoxic conditions for 0 to 168h with the presence of CD47. The level of HIF1α in nucleus was determined by immunoblotting. Data is analyzed using one-way ANOVA followed by Tukey multiple range test and expressed as mean ± SD. ***p<0.001, ns, non-significant.

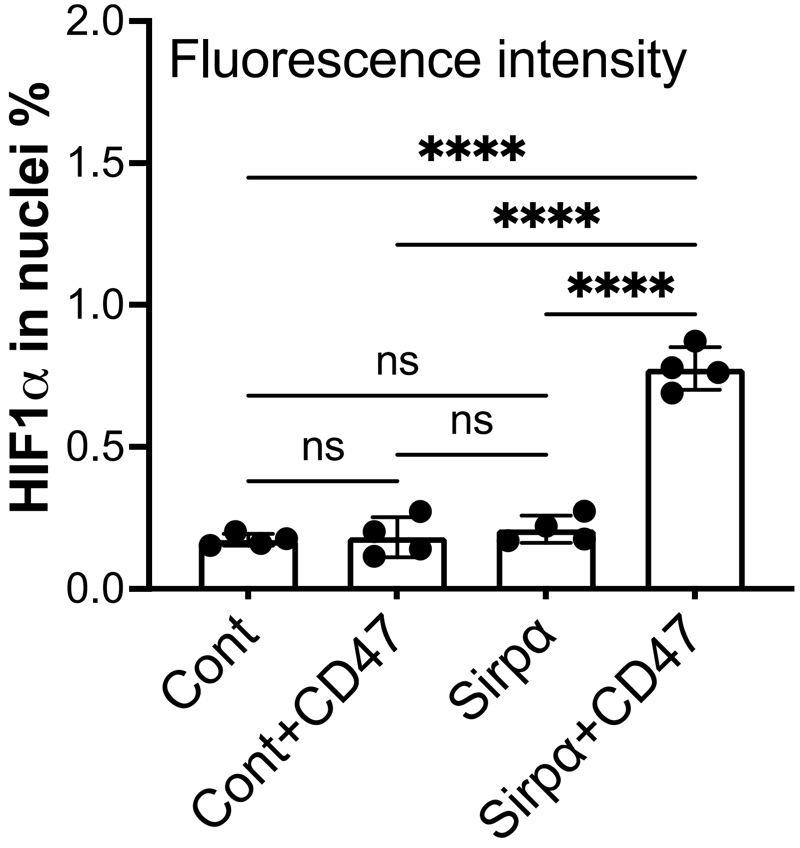

sFigure 21. Macrophages were incubated in hypoxic condition. The fluorescence intensity of HIF1α in the Sirpα overexpressed macrophages nuclei with or without CD47, Lentiviruses control-treated macrophages were used as a control (n=4). Data is analyzed using one-way ANOVA followed by Tukey multiple range test and expressed as mean ± SD , ****p<0.0001. ns, non-significant.

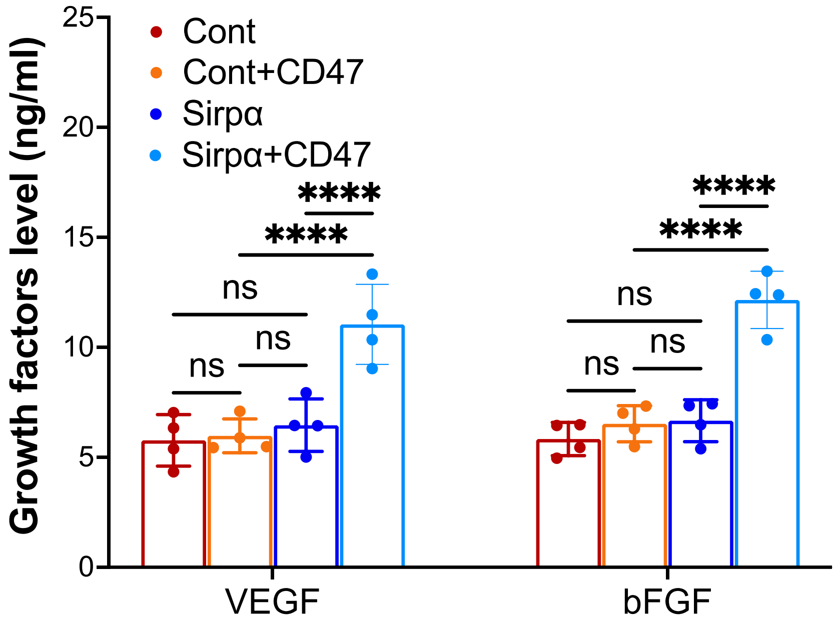

sFigure 22. Macrophage were collected from the adductor muscles of WT mice. The level of growth factors in the Sirpα overexpressed macrophages with or without the presence of CD47 under hypoxic condition. Cont: Lentiviruses control-treated macrophage without the presence of CD47, Cont CD47: Lentiviruses control-treated macrophage with the presence of CD47, Sirpα: Sirpα overexpressed macrophage without the presence of CD47, Sirpα CD47: Sirpα overexpressed macrophage with the presence of CD47. Data is analyzed using one-way ANOVA followed by Tukey multiple range test and expressed as mean ± SD , ****p<0.0001. ns, non-significant.

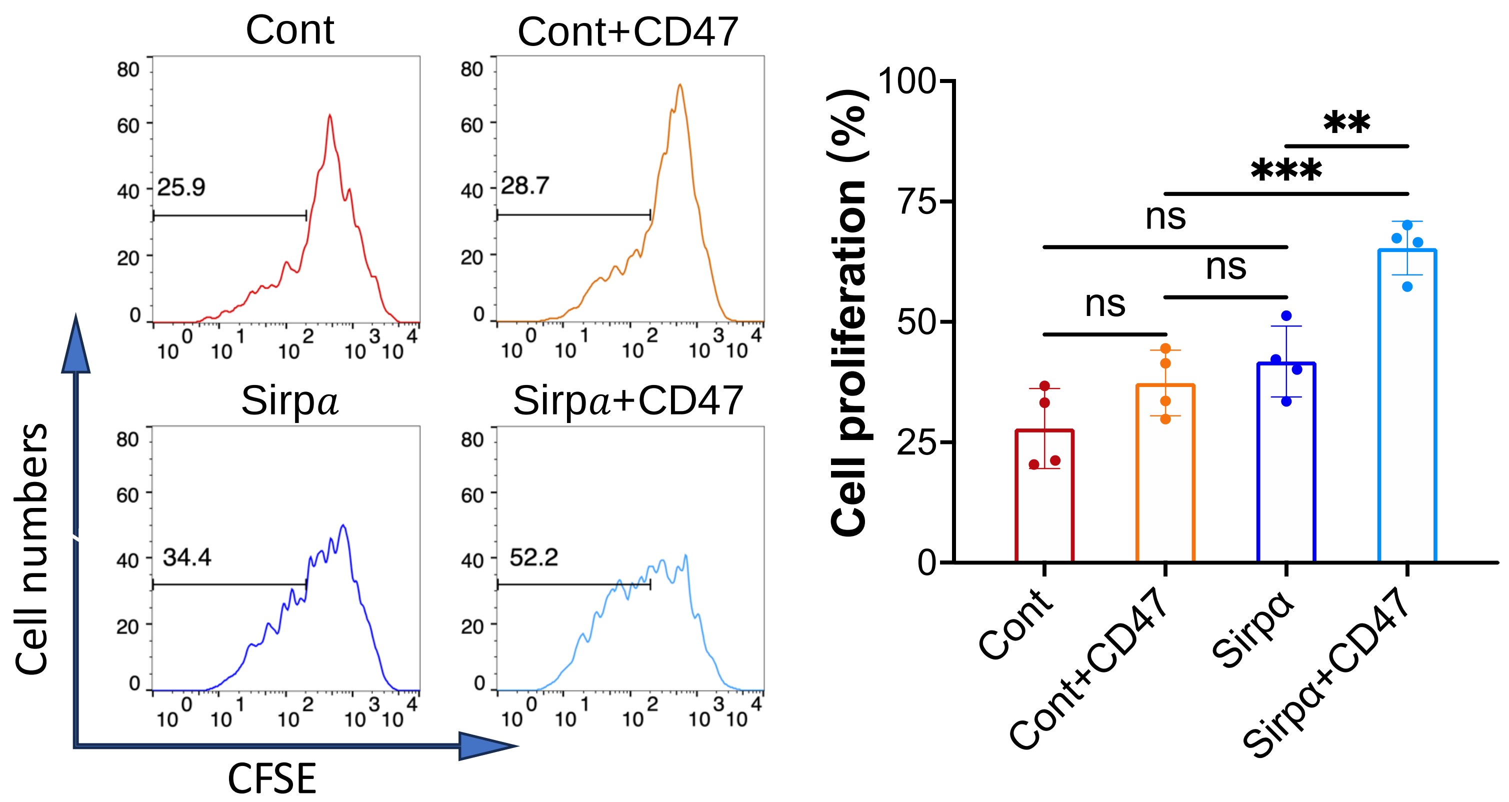

sFigure 23. CMVECs were treated with various conditioned medium (CdM) from the macrophages for 4 days. Cell proliferation assay of the CMVECs were performed. Data is analyzed using one-way ANOVA followed by Tukey multiple range test and expressed as mean ± SD , **p<0.01 and ***p<0.001. ns, non-significant. Total 5000 cells were analyzed.

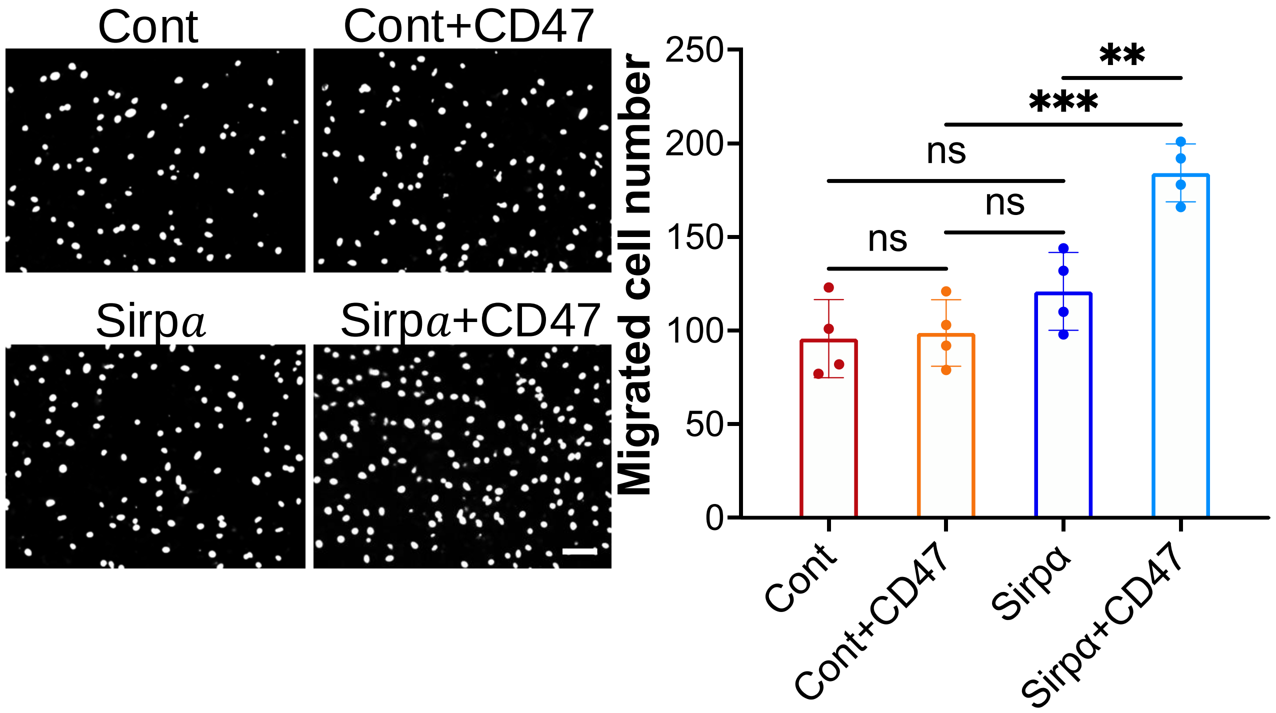

sFigure 24. CMVECs were treated with various conditioned medium (CdM) from macrophages. Cell migration assay of the CMVECs were performed, scale bar: 100 µm. Data is analyzed using one-way ANOVA followed by Tukey multiple range test and expressed as mean ± SD , ***p*<0.01 and ****p*<0.001. ns, non-significant.

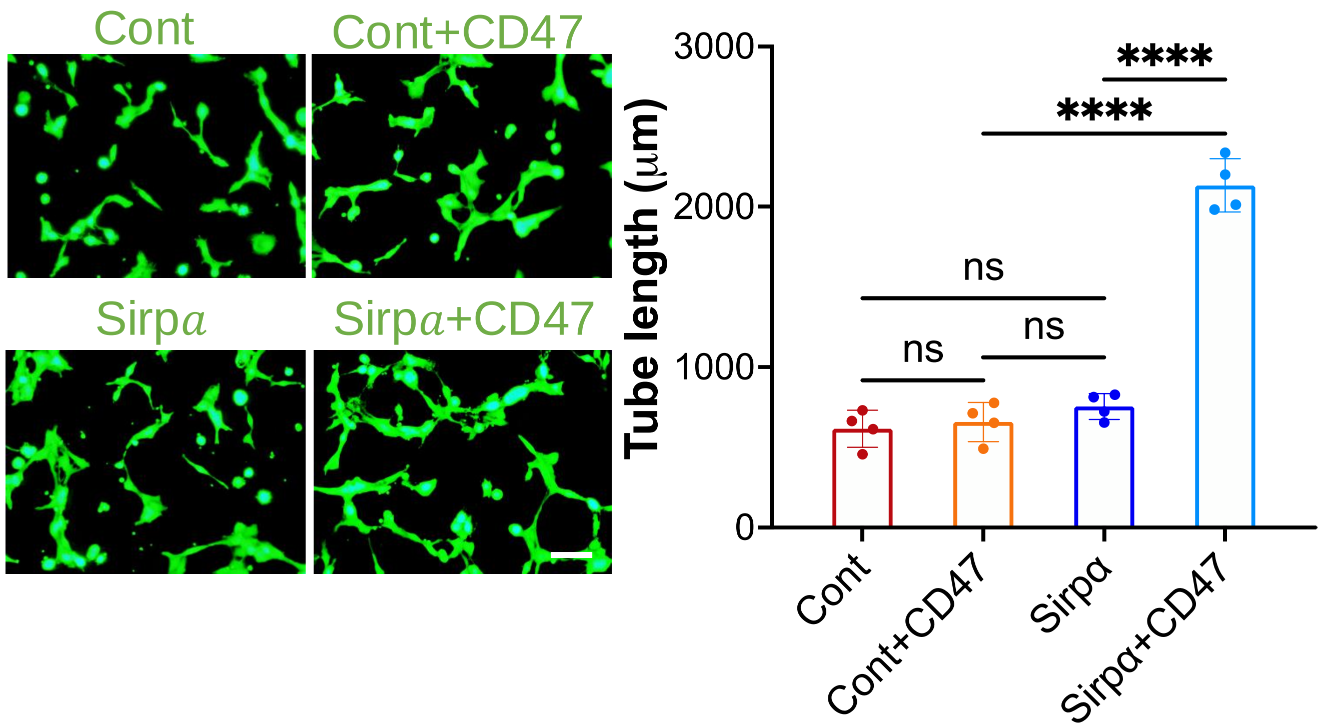

sFigure 25. CMVECs were treated with various conditioned medium (CdM) from macrophages. Tube formation assay of the CMVECs were performed, scale bar: 50 µm. Data is analyzed using one-way ANOVA followed by Tukey multiple range test and expressed as mean ± SD , ****p<0.0001. ns, non-significant.

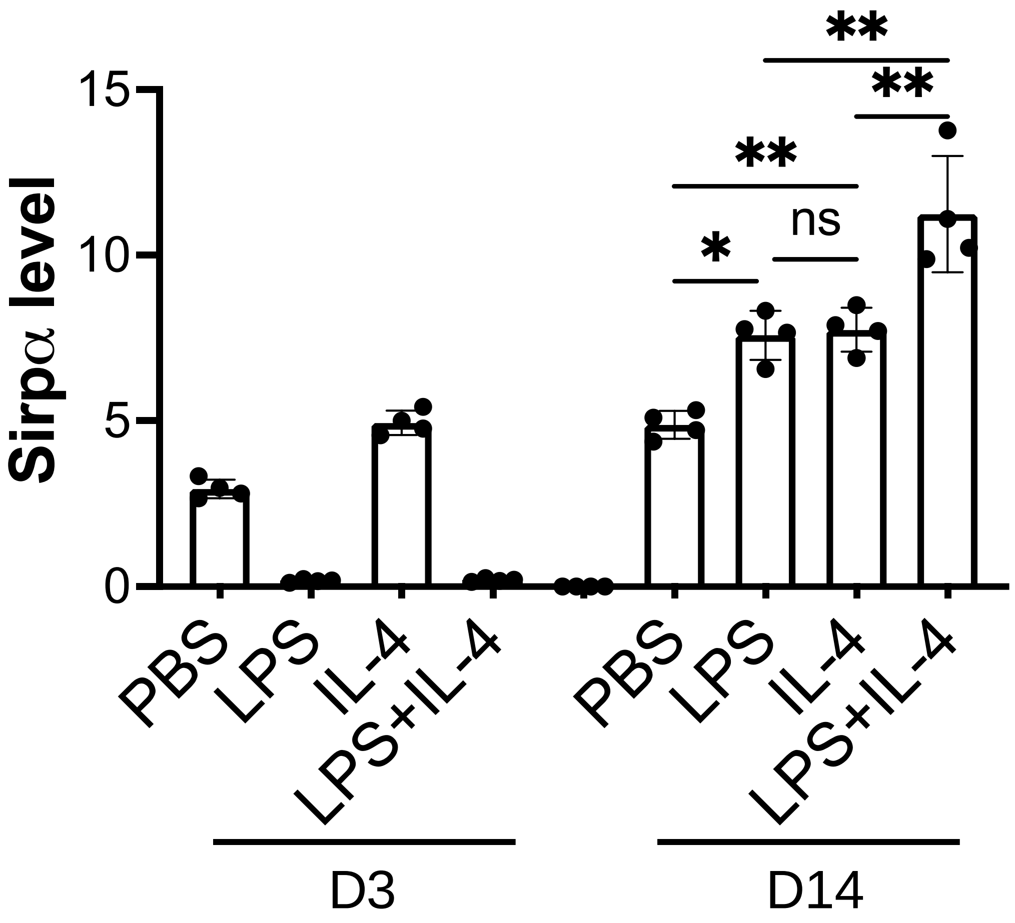

sFigure 26. Quantitative analysis of Sirpα level in the infarcted heart. Data is analyzed using one-way ANOVA followed by Tukey multiple range test and expressed as mean ± SD , *p<0.05 and **p<0.01. ns, non-significant.

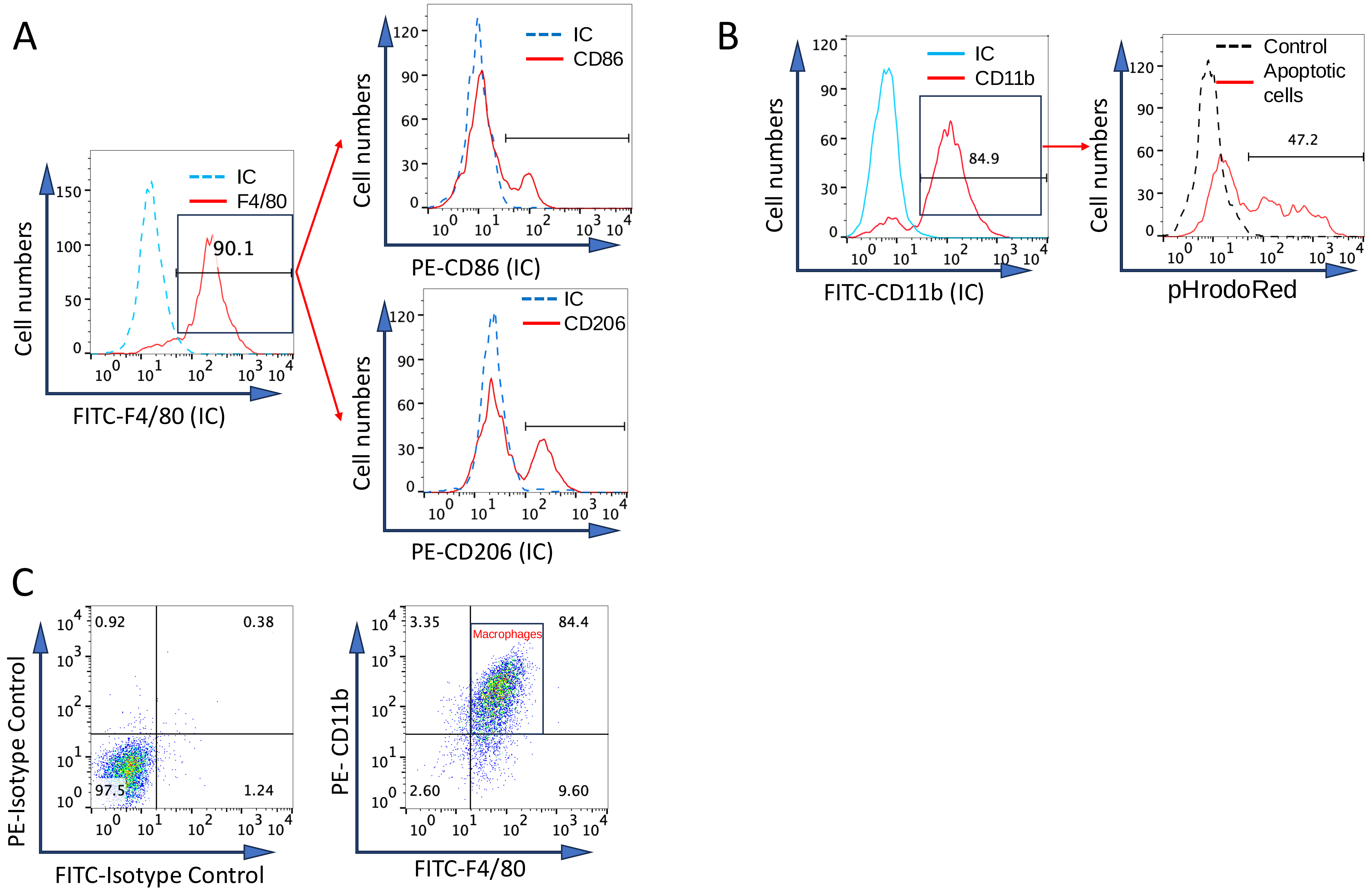

sFigure 27. A and B, Gating strategies of flow cytometry. **C,** The purity of isolated tissue macrophages. IC, isotype control.

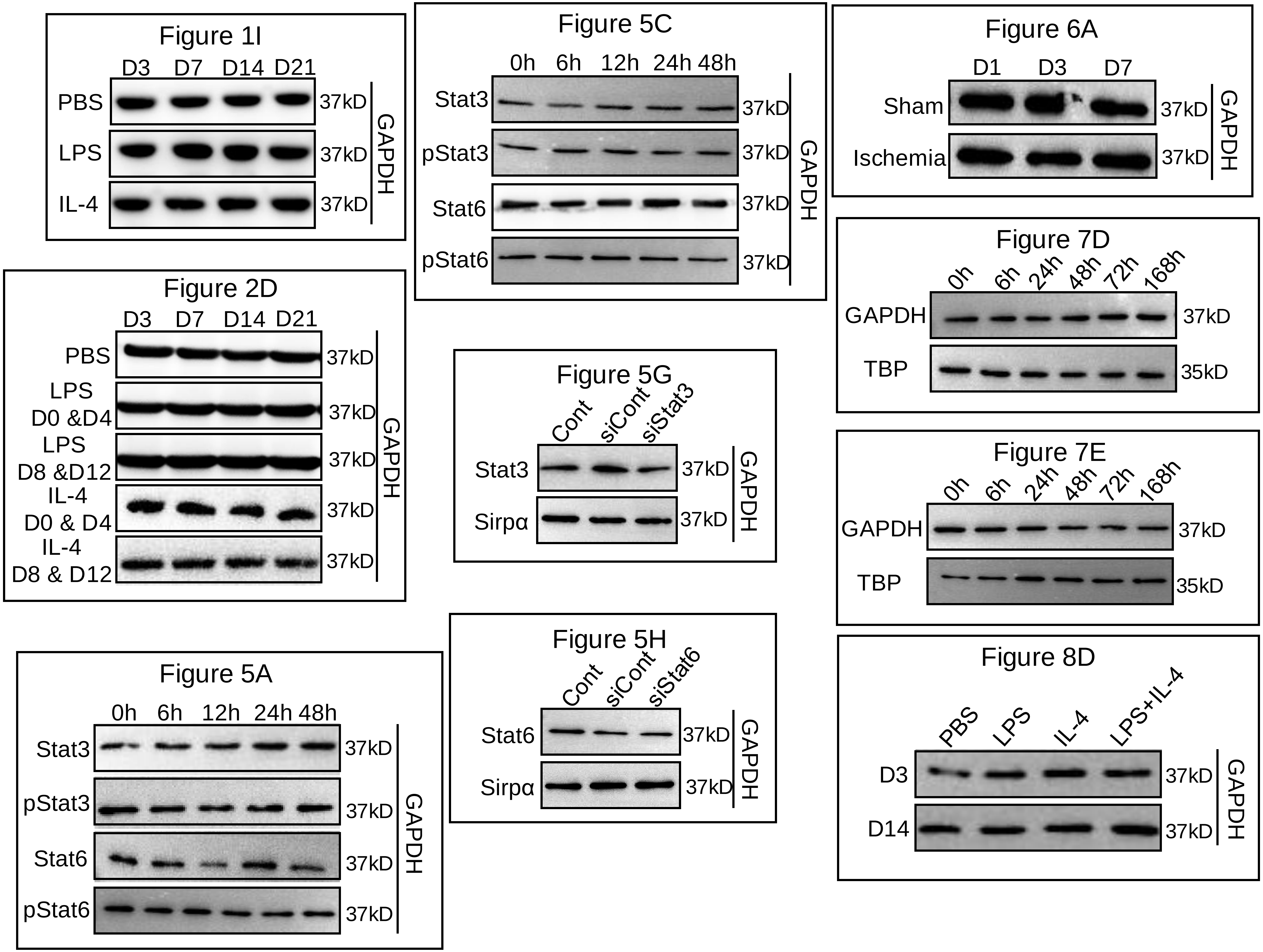

sFigure 28. GAPDH (37kD) or TBP (35KD) was used as a loading control respectively.
